# Chemogenomics for drug discovery: clinical molecules from open access chemical probes

**DOI:** 10.1039/d1cb00016k

**Published:** 2021-03-26

**Authors:** Robert B. A. Quinlan, Paul E. Brennan

**Affiliations:** Target Discovery Institute, Nuffield Department of Medicine, University of Oxford Old Road Campus Oxford OX3 7FZ UK paul.brennan@cmd.ox.ac.uk; Alzheimer's Research (UK) Oxford Drug Discovery Institute, Nuffield Department of Medicine, University of Oxford Oxford OX3 7FZ UK

## Abstract

In recent years chemical probes have proved valuable tools for the validation of disease-modifying targets, facilitating investigation of target function, safety, and translation. Whilst probes and drugs often differ in their properties, there is a belief that chemical probes are useful for translational studies and can accelerate the drug discovery process by providing a starting point for small molecule drugs. This review seeks to describe clinical candidates that have been inspired by, or derived from, chemical probes, and the process behind their development. By focusing primarily on examples of probes developed by the Structural Genomics Consortium, we examine a variety of epigenetic modulators along with other classes of probe.

## Abstract 2

Chemical probes are of great use for investigating target safety, viability, and translation. Alongside this, they act as useful structural templates to inspire drug discovery.

## Graphical abstract

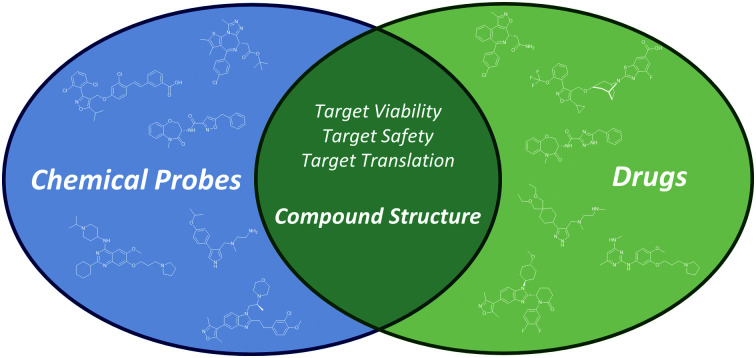

## Introduction

Progress in the understanding of cellular and disease biology has advanced our knowledge of protein function beyond solely catalysis.^1–5^ We have begun to better characterise the extra-catalytic roles of proteins and how these contribute to disease pathology, whether as readers of epigenetic marks, as molecular chaperones, or scaffolding proteins. To that end, non-destructive means of target validation and investigation are useful to examine the precise role of a protein in a cellular process. Where the use of RNA inhibition (RNAi) or CRISPR gene editing precludes this, chemical probes are an invaluable tool for rapid onset, reversible, and domain specific protein inhibition.^6–8^ They permit the evaluation of a particular role a protein plays, whilst leaving other interactions intact. In combination with techniques such as RNAi, they paint a complete picture of a protein's role within a cell.^7^

Chemical probes are defined by four main criteria:^6,9,10^

1. A minimal *in vitro* potency of less than 100 nM.

2. Greater than 30-fold selectivity over sequence-related proteins.

3. Profiled against an industry standard selection of pharmacologically relevant targets.

4. On-target cellular effects at greater than 1 μM.

Their utility in interrogating the function of a protein and thus its relevance^11^ as a drug target has led a consortium of industrial and academic researchers to establish a collaboration for the development of probes for the entire proteome.^12^ Target 2035 aims to translate the advances made in genomics^13^ to advances in the clinic, specifically new small-molecules for the treatment of disease. One advantage of chemical probes often touted is that they are more likely to mimic the pharmacology of a drug.^7^ Whilst not designed with drug-like characteristics in mind (for example absorption, distribution, metabolism, and excretion (ADME) properties),^7^ there is nevertheless a belief that probes can provide a small-molecule starting point to accelerate the drug discovery process. In this review, we highlight some small-molecule chemical probes that have both inspired and mirrored clinical candidates, with a focus on epigenetic modulators. We describe the target and development of the probe itself, the medicinal chemistry optimisation that led to the eventual clinical candidate. We also include an estimate of the relative drug-likeness of each compound calculated using the Molsoft drug-likness and molecular property prediction tool. A higher score indicates greater drug-likeness with drugs distributed around a score of one, and non-drugs at zero.^14^

## BET bromodomains

Bromodomains (BRDs) are a class of epigenetic reader domains that recognise acetylated lysine (KAc) residues,^15^ a reversible post-translational modification with a key role in regulating transcription.^16^ A subset of BRDs have also been shown to recognise propionylated, butyrylated, and crotonylated lysines,^17,18^ demonstrating the importance of these domains in interpreting the chromatin landscape. Evolutionarily conserved, they form part of diverse nuclear proteins and complexes.^19^ The bromo- and extra C-terminal (BET) subfamily of bromodomains is perhaps the best studied and validated in a disease context.^4,20,21^ Its members (BRD2, BRD3, BRD4, and BRDT) have been implicated in a variety of disease processes, including cancer, viral infection, and inflammation.^22–25^

### The probe: **(+)-JQ1**

**Fig. 1:**
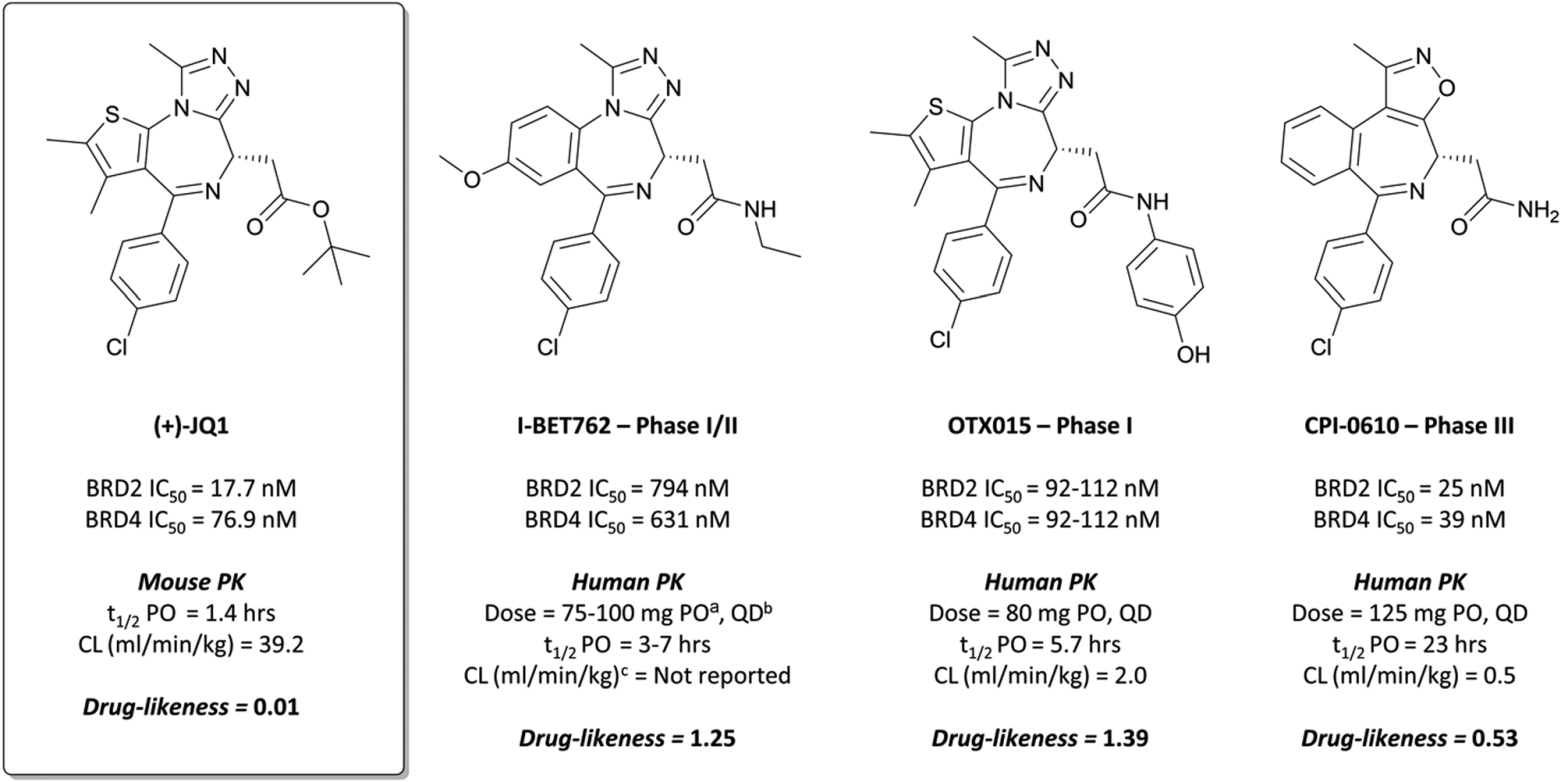
Chemical probe **(+)-JQ1** and structurally derived clinical candidates: **I-BET762**, **OTX015**, and **CPI-0610**. Their potency against relevant BET bromodomains is presented, along with pharmacokinetic (PK) data where available, and their clinical status. Also included is a relative drug-likeness score to highlight the changes in drug-likeness moving from probe to drug. ^*a*^ PO = *per os*, by mouth. ^*b*^ QD = *quaque die*, once daily. ^*c*^ Assume 70 kg bodyweight for humans.

Disclosed just months apart, the first pan-BET inhibitors **(+)-JQ1**^26^ and **I-BET762**^27^ (Fig. 1) represented key milestones in the targeting of BET proteins. Inspired by a triazolothienodiazepine scaffold patented in 2009,^28^**(+)-JQ1** was developed following molecular modelling of potential ligands against the bromodomain of BRD4. It was a potent inhibitor of both bromodomains of BRD4 (*K*_D_(BRD4(1)) = 50 nM, *K*_D_(BRD4(2)) = 90 nM by isothermal titration calorimetry (ITC)), with similar potency against both bromodomains of BRD3. It showed approximately three-fold weaker binding against BRD2 and BRDT. Pan-BET inhibition using **(+)-JQ1** has shown anti-proliferative effects against a multitude of haematological and solid malignancies, including breast,^29,30^ colorectal,^31^ and brain cancers,^32,33^ as well as multiple myeloma (MM),^34,35^ leukaemia,^36–38^ and lymphoma.^35,38,39^**(+)-JQ1** was key to establishing the mechanistic significance of BET inhibition, however was unsuitable for clinical progression due to its short half-life^26^ and thus its required dose concentrations being above tolerable levels *in vivo.*^40^

#### **I-BET762**/GSK525762/molibresib

**Fig. 2:**
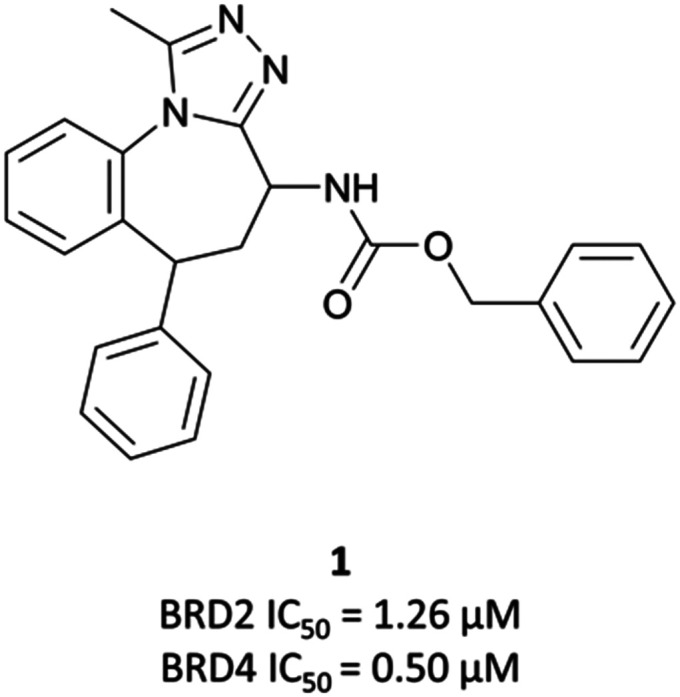
Initial hit **1** in a screen for compounds that upregulated ApoA1 expression.

**I-BET762** was identified following a screen of compounds that upregulated the Apolipoprotein A1 (ApoA1) gene as a proxy for BET inhibition,^27^ leading to the identification of an initial hit (Fig. 2, **1**) with an EC_50_ of 440 nM for the induction of ApoA1.^41^**1** shared a markedly similar triazolodiazepine-based structure to **(+)-JQ1**, including the vectors off the scaffold. Pull-down assays identified the molecular targets of **1** to be the BET proteins BRD2, -3, and -4 (IC_50_s (fluorescence polarisation, FP) = 1.25 μM, 631 nM, and 501 nM respectively).^42^

Optimisation efforts focused on improving potency, target selectivity, physiochemical properties, and compound stability.^41^ Previous studies on the stability of triazolobenzodiazepines had identified problems with ring-opening under acidic conditions, precluding oral dosing.^43^ The authors observed this with some analogues of **1**, measuring very short half-lives (<1 h) when the compounds were suspended in buffer at pH = 2.0. They confronted this by eliminating the nitrogen at the 3-position of the benzodiazepine ring of **1**, replacing the amide with the acetamide of **I-BET762**. This had the added benefit of simplifying the enantioselective synthesis of further analogues, as aspartic acid could be used as a chiral precursor. The ethylacetamide of **I-BET762** in lieu of the phenylcarbamate of **3** lowered both the log *P* and molecular weight (MW) to further improve the oral profile. Other improvements came from SAR that introduced the methoxy- and chloro-substituents on their respective phenyl rings to yield **I-BET762** (IC_50_ (FP) = 794 nM, 398 nM, 631 nM for BRD2, -3, and -4 respectively).

Whilst structurally very similar to **(+)-JQ1**, **I-BET762** displayed more favourable pharmacokinetic (PK) properties, with good solubility and improved half-life^41,44^ reflected in an improved drug-likeness. **I-BET762** showed no appreciable activity in safety assays and was selective against other representative bromodomains. It induced growth inhibition in *in vivo* models of nuclear protein in testis (NUT) midline carcinoma,^44^ MM,^45^ and prostate cancer.^46^ In 2012, **I-BET762** was advanced to the clinic for the treatment of NUT carcinoma and other solid tumours.^44^ Target engagement was observed with once-daily dosing, with several patients experiencing clinical benefit. Despite that, **I-BET762** showed rapid elimination (*t*_1/2_ = 3–7 hours) and transient responses, thought to be due to the activation of resistance mechanisms to BET inhibition. Nevertheless, it is currently under clinical investigation for the treatment of acute myeloid leukaemia (AML) (NCT01943851), with manageable adverse events and some objective responses observed.^47^ It is also being evaluated as part of a combination therapy for breast cancer (NCT02964507, expected completion August 2021)^48^ and prostate cancer (NCT03150056, expected completion August 2021).^49^

#### **OTX015**/MK-8628

**OTX015** (Fig. 1) is another triazolothienodiazepine-based BET inhibitor and almost structurally indistinct from **(+)-JQ1**, though with alterations enough to improve drug-likeness substantially.^50,51^ Initially identified in a screen for inhibitors of cell adhesion, it was subsequently tested against the BET proteins and discovered to be a potent inhibitor, with IC_50_ values of 92–112 nM in a fluorescence resonance energy transfer (FRET) assay. It induced significant *in vitro* and *in vivo* growth inhibition in a number of cancer cell lines derived from both solid and haematological malignancies, including breast cancer,^52^ lung cancer,^53^ glioblastoma,^54^ and leukaemia.^55^

After preclinical models demonstrated good oral bioavailability and PK properties,^53,56^**OTX015** entered the clinic as a treatment for a variety of haematological malignancies. It showed dose-limiting toxicities (DLTs) in patients with lymphoma and MM,^57^ and in a study for patients with leukaemia.^58^ These findings informed a once-daily dosing regimen that led to some partial responses, however it was rapidly eliminated (*t*_1/2_ = 5.7 h) and there was no evidence of clinical activity that met objective response criteria the majority of patients. It was also evaluated against some solid tumours (prostate cancer, NUT midline carcinoma, and non-small-cell lung cancer (NSCLC)),^59^ however displayed DLTs alongside a lack of efficacy. The same lack of efficacy was observed in a phase I trial of **OTX015** against glioblastoma (NCT02698176). As a result of these collective failures, Merck terminated the OTX015 program (NCT02698189).

#### CPI-0610

**Fig. 3:**
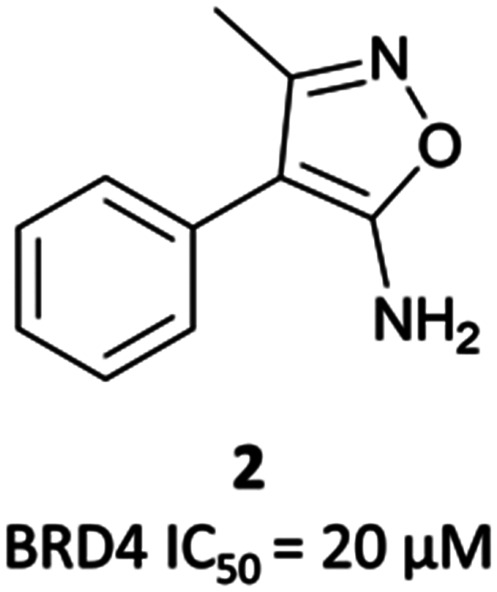
Aminoisoxazole fragment hit **2** identified from a screen against the first bromodomain of BRD4.

Researchers from Constellation Pharmaceuticals drew direct inspiration from **(+)-JQ1** during the development of their own BET inhibitor, **CPI-0610** (Fig. 1).^60^ A thermal shift assay against BRD4(BD1) initially identified an aminoisoxazole fragment (Fig. 3, **2**) which bound to BRD4 in a similar mode to the triazole portion of **(+)-JQ1** and **I-BET762**, the isoxazole mimicking the *N*-acetyllysine motif of the histone.^26,27^ They hypothesised that the introduction of a azepine ring, analogous to **(+)-JQ1**, would constrain the fragment and provide a vector from which to interact with the hydrophobic region of the binding pocket.

**Fig. 4:**
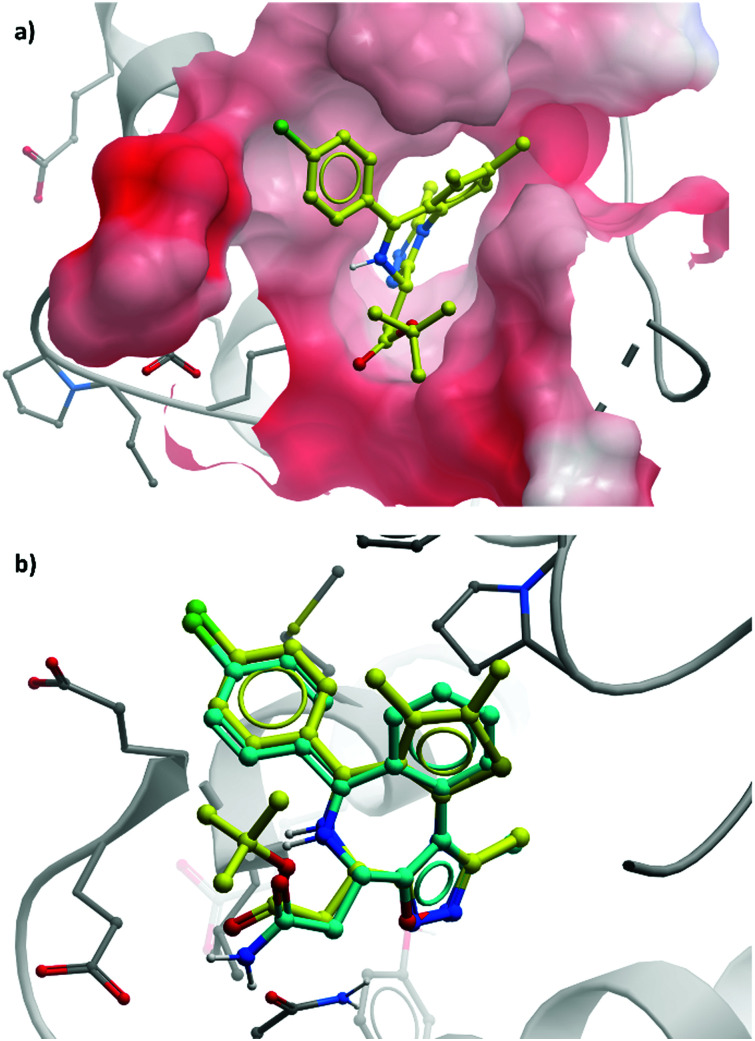
(a) **(+)-JQ1** bound to the first bromodomain of BRD4, extending into the acetyllysine pocket (PDB: 3MXF); (b) the binding mode of **CPI-0610** (cyan) overlaid with **(+)-JQ1** (yellow), showing significant overlap (PDB:5HLS).

**2** binds to BRD4(BD1) with an IC_50_ (FRET) of 20 μM, which improved to 440 nM following the introduction of an azepine ring. Interestingly, this was more potent than the corresponding triazole compound, thought to be because it better engaged in hydrogen bonding interactions with Asn140 and Tyr97 in the binding site. Substitution on the methylene bridge of the azepine ring found that the (*S*)-enantiomer of an *N*-unsubstituted acetamide gave the most potent compound (**CPI-0610**) with a BRD4(1) IC_50_ (FRET) of 39 nM, binding in identical fashion to **(+)-JQ1** (Fig. 4b) and with slight improvement in drug-likeness. Modulation of the chlorophenyl group gave no advantage, and whilst more potent compounds were synthesised through substitution on the benzo ring, these improvements in potency were not observed in cells. **CPI-0610** was selective against non-BET bromodomains, as well as against targets in a CEREP express panel of 50 GPCRs, ion channels and transporters, and showed negligible inhibition of CYP450.

**CPI-0610** displayed favourable PK in various species, with low clearance, moderate half-life, and good oral bioavailability.^60^ In a mouse xenograft model of AML, it induced a dose-dependent reduction in MYC mRNA levels, as well as significant tumour growth inhibition, with no associated weight loss observed. It also induced growth inhibition and improved survival in a mouse xenograft model of MM.^61^ These findings led Constellation to advance **CPI-0610** to early phase clinical studies for the treatment of haematological malignancies. Phase 1 evaluation in patients with relapsed or refractory lymphoma^62–64^ showed that once-daily oral dosing was well tolerated and induced antitumour responses, as well as dose-dependent decreases in the expression of BET target genes. **CPI-0610** demonstrated good oral bioavailability and stability (*t*_1/2_ = 23 h).^62^

Following these results, **CPI-0610** was initiated in a phase II trial for the treatment of myelofibrosis.^65,66^ The trial had two arms, with **CPI-0610** as a monotherapy for myelofibrosis (NCT04603495, expected completion September 2023), or in combination with the Janus kinase (JAK) 1/2 inhibitor ruxolitinib (NCT02158858, expected completion December 2021).^67^ Its use as part of a combination therapy derives from the results of a previous study that showed combining JAK and BET inhibition (with ruxolitinib and **(+)-JQ1**) in mice models of myelofibrosis led to a marked reduction in disease burden and the serum levels of inflammatory cytokines *in vivo*.^68^ Thus far, the combination has been well tolerated and objective responses have been observed in all evaluable patients,^66^ indicating promise for the use of **CPI-0610** in combination with ruxolitinib for the treatment of myelofibrosis.

### The probe: **PFI-1**

In the search for life beyond triazolodiazepine BET inhibitors, several groups described orthogonal chemotypes that also bound to BET proteins.^69–72^ In particular, 3,5-dimethylisoxazole- and quinazolinone-based fragments were found to mimic the *N*-acetyllysine binding mode of the histone peptide, forming a productive hydrogen bond with Asn140 in the BRD4(BD1) binding pocket (*vide supra* the effective use of an isoxazole fragment in the development of **CPI-0610**). Inspired by these findings, a collaboration between scientists at Pfizer and the SGC used a fragment-led approach to discover **PFI-1** (Fig. 7),^73^ a structurally novel pan-BET inhibitor.

**Fig. 5:**
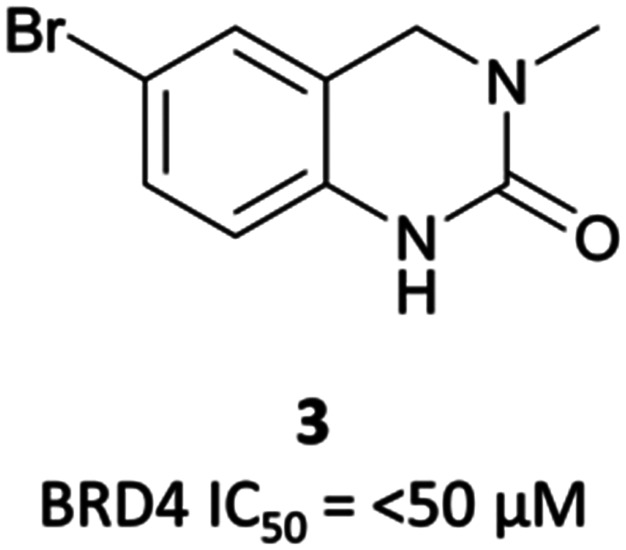
Quinazolinone fragment **3**.

Taking inspiration from previous work by groups from GSK^71^ and the SGC,^72^ the authors chose fragments based on a 4-dihydro-3-methyl-2(1*H*)-quinazolinone scaffold as their starting point, identifying a brominated derivative (Fig. 5, **3**) as a promising initial hit.^73^ A crystal structure of **3** in complex with BRD4(BD1) showed that the cyclic urea motif was indeed engaging in a hydrogen bonding interaction with Asn140, and that the rest of the scaffold was interacting with a variety of lipophilic residues in the protein. Interrogation of the crystal structure suggested the introduction of a kink in the molecule, using the 6-bromo moiety as a synthetic handle, would enable engagement of the lipophilic WPF shelf at the opening of the binding pocket. This was effectively achieved through the introduction of an arylsulfonamide group off this position, resulting in **PFI-1** that had an IC_50_ (alphascreen) of 220 nM 98 nM for BRD2(BD1) and (BD2) respectively.^74^ Thermal shift assays showed that **PFI-1** was selective for the BET bromodomains over other representative bromodomains, and assessment against other pharmacologically relevant targets selectivity over a panel of 65 GPCRs, ion channels, and kinases.^73^

**PFI-1** induced growth inhibition and apoptosis in a dose-dependent manner in leukaemia cells,^74^ and also downregulated Aurora B kinase expression, which is implicated in a number of cancers, including leukaemia.^75^ It displayed poor PK properties in rats, with poor oral bioavailability, high clearance, and a short half-life. Nevertheless, its selectivity, along with its on-target and cellular potency, identified it as an effective chemical probe for interrogation of the BET bromodomains.

#### **ABBV-075**/mivebresib

**Fig. 6:**
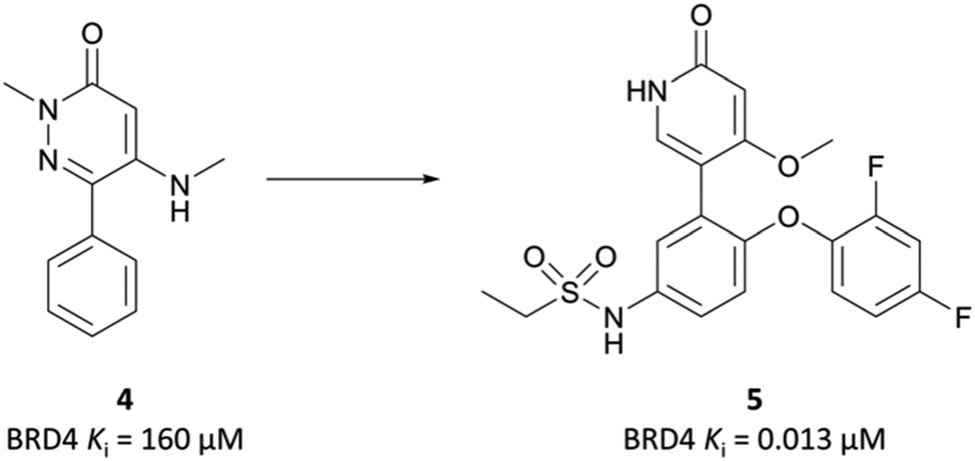
Phenylpyridazinone fragment **4** around which SAR exploration led to **5**.

**Fig. 7:**
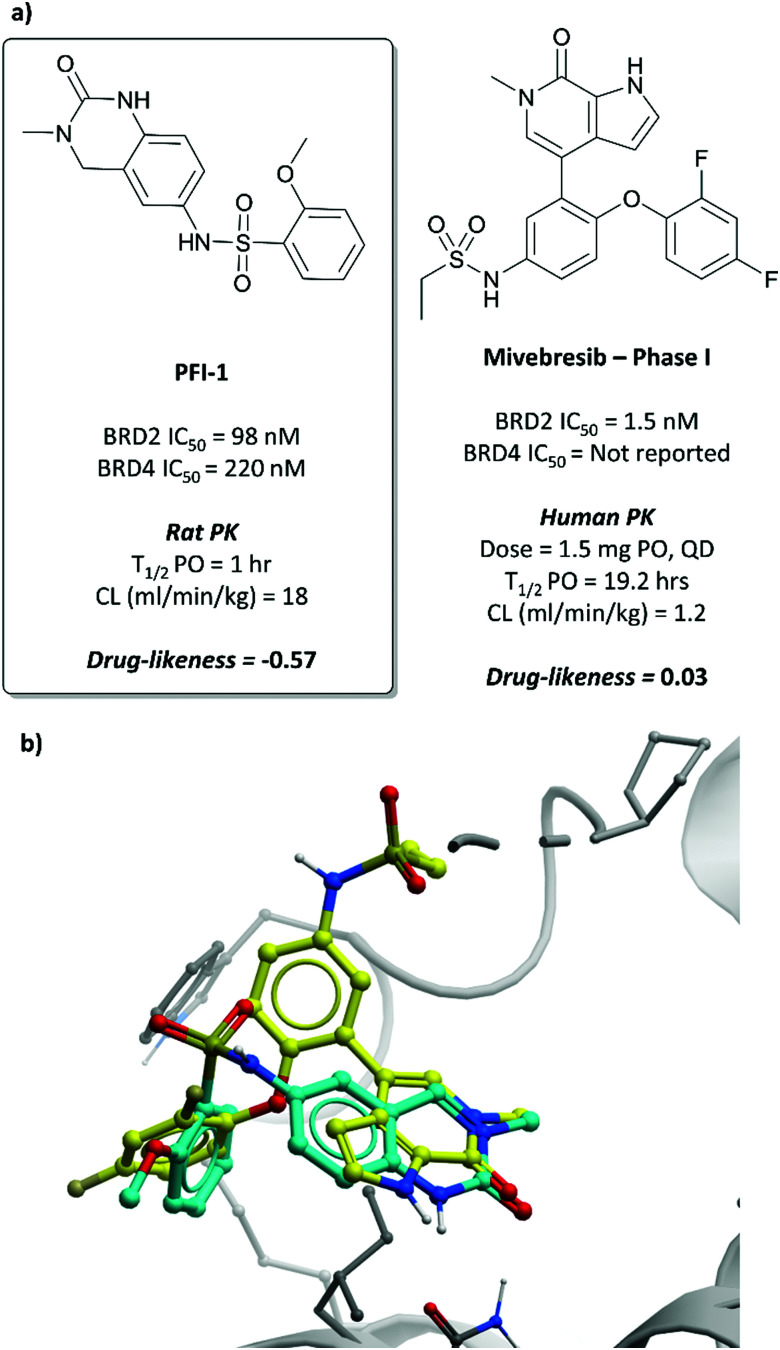
(a) Probe **PFI-1** and clinical molecule **mivebresib**; (b) overlaid crystal structures of **PFI-1** (cyan) and **mivebresib** (yellow) bound to the first bromodomain of BRD4. The quinazolinone of **PFI-1** and pyrrolopyridone of **mivebresib** align, mimicking an acetylated lysine. The phenylether of **mivebresib** also occupies the same vector as the arylsulfonamide of **PFI-1** (PDB: 4E96 and 5UVW).

A similar fragment-based approach led to the discovery of **mivebresib** (Fig. 7), following the initial discovery of a similar acetyllysine mimic to that which led to **PFI-1**.^76,77^ An NMR screen identified a phenylpyridazinone fragment (Fig. 6, **4**), a weak binder which had *K*_*i*_ (FRET) against BRD4(BD2) of 160 μM. Examination of the crystal structure of **4** bound to BRD4(BD2) and comparison to that of **(+)-JQ1**^26^ identified key vectors for SAR exploration. Introduction of a phenyl ether to the phenyl ring of **4** enabled productive engagement with the WPF shelf, in a similar manner to the strategy employed in the discovery of **PFI-1**. A switch from a pyridazinone to a pyridone core, as well as substitution of the methylamine group with a methoxy group led to further improvements in potency. These were built on with the introduction of an ethylsulfonamide *para* to the phenyl ether on the phenyl ring of **4**. This new derivative was a potent binder (*K*_*i*_ FRET (BRD4(BD2) = 4.4 nM), however underwent significant oxidative metabolism in microsomal stability assays. Fluorination of the phenyl ether moiety led to **5** (Fig. 6), which remained a potent binder but showed substantially improved stability in microsomes. **5** also potently inhibited the proliferation of MX-1 cells, a breast cancer cell line (EC_50_ = 47 nM). No selectivity was observed over the other BET proteins, however **5** was highly selective against other representative bromodomains, with only weak inhibition observed at 1–2 μM for four related proteins.

Because of its favourable PK properties, namely good oral bioavailability (*F*% (mouse) = 63), **5** was chosen for further evaluation in a mouse xenograft model of MM.^76^ Oral dosing led to significant dose-dependent tumour growth inhibition, with acceptable weight loss observed. Enthused by these results, the authors decided to evaluate whether further gains in potency could be achieved through a bidentate interaction that would engage the NH_2_ group of Asn433 in the BRD4(BD2) binding pocket.^77^ By transforming the pyridone core to a bicyclic pyrrolopyridone (**mivebresib**), they were able to form productive hydrogen bonds to both the carbonyl and the NH_2_ of Asn433. **Mivebresib** was a potent binder, with a *K*_*i*_ (FRET) against BRD4(BD2) of 1.5 nM and remained potent in cellular assays (EC_50_ (MX-1 proliferation) = 13 nM). Whilst displaying poor drug-likeness, it was substantially improved relative to **PFI-1**. It was selective against a panel of 79 pharmacologically relevant targets and exhibited favourable PK and drug-like properties, including low clearance in human liver microsomes.^78,79^

In a mouse xenograft model of AML, **mivebresib** was dosed orally at 1 mg kg^−1^ for 25 days and achieved 99% tumour growth inhibition, with acceptable weight loss observed. Further PK characterisation across species identified this compound as a clinical candidate, leading to its evaluation as a treatment for patients with relapsed/refractory solid tumours, including breast and prostate cancer, as well as melanoma.^80^**Mivebresib** was well tolerated, with some DLTs and a half-life of 16.1–19.9 h. Despite these favourable characteristics, very little efficacy was observed beyond stable disease for a modest subset of patients. Preclinical studies demonstrated that this lack of efficacy may be improved by its use as a combination therapy,^81^ and so a phase I trial evaluating **mivebresib** in combination with the BCL_2_ inhibitor **navitoclax** is ongoing (NCT04480086, expected completion July 2024).

#### CBP/p300

The cyclic-AMP response element binding (CREB) protein binding protein (CBP) and E1A binding protein (p300) are ubiquitously expressed proteins involved in a variety of cellular processes, acting as lysine acetyltransferases and transcriptional co-factors.^82–86^ Both are modular proteins, containing several domains that bind to transcription factors,^87–90^ as well as a bromodomain and a histone acetyltransferase (HAT) domain.^91,92^ The structure of the bromodomains is highly conserved, with a 96% sequence homology between the two.^93^ Acetyllysine marks on these transcription factors are responsible for the recruitment of CBP/p300 *via* their bromodomains, as is the case with CBP and the tumour suppressor protein p53.^89,90^ Paradoxically, CBP/p300 also interact with a variety of oncogenes *via* their bromodomains,^82^ demonstrating their ability to exert context-dependent transcriptional control.

Whilst involved in the suppression of tumour formation, p53 has also been shown to mediate excessive apoptotic death in normal cells following cancer therapy.^94^ As well as this, its overactivity has been implicated in a variety of neurodegenerative and cardiovascular diseases,^95–100^ suggesting its inhibition may be of therapeutic value.

### The probe: **CBP-30**

The potential benefit of blocking the interaction of CBP with p53 and thus preventing the transcription of p53-mediated genes^101,102^ led researchers at the SGC to embark on the development of **CBP-30** (Fig. 9),^93^ a potent and selective inhibitor of the CBP/p300 bromodomains.

**Fig. 8:**
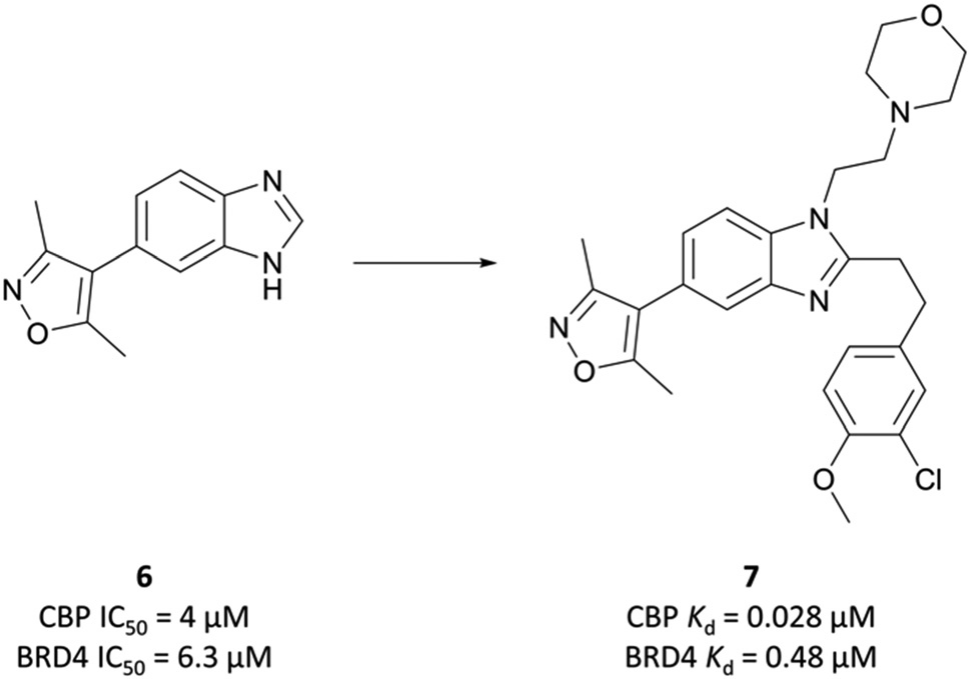
Starting isoxazole fragment **6** and the subsequently derived **7**, with improved potency for CBP and selectivity over BRD4.

The starting point was a non-selective isoxazole fragment (Fig. 8, **6**) that inhibited CBP/p300 and BRD4 with similar micromolar potency.^103^ Subsequent derivatives would be designed to achieve selectivity for CBP/p300 over the BET bromodomains. Synthesis of a variety of analogues of **6** led to the discovery that *N*1- and *C*2-substitution increased the potency to sub-micromolar levels against CBP, inducing some selectivity over BRD4(BD1). Optimisation of both the *N*1-amine substituent and the *C*2-aryl component through introduction of morpholine and 3-chloro-4-methoxyphenyl moieties respectively, led to compound **7** (Fig. 8). **7** had a *K*_D_ (ITC) of 28 nM and 480 nM against CBP and BRD4(BD1) respectively, a selectivity of 17-fold.

**Fig. 9:**
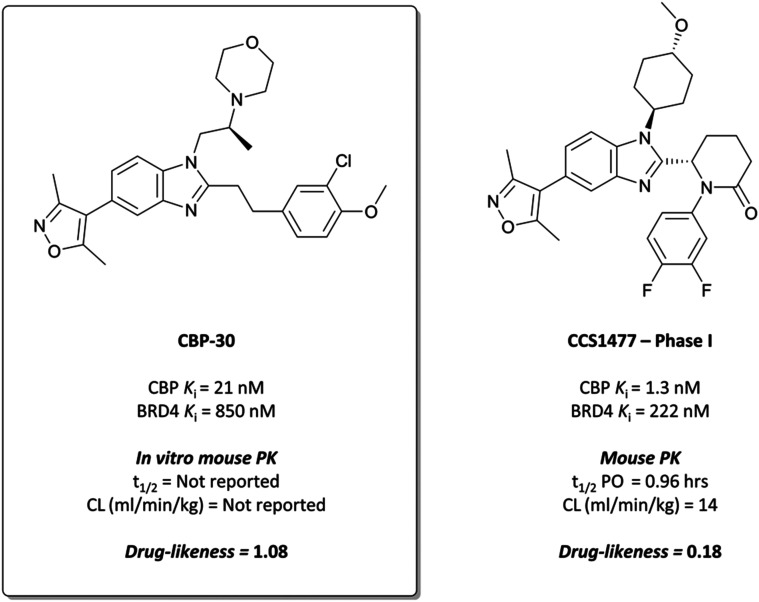
Chemical probe for CBP/p300 **CBP30**, and clinical candidate **CCS1477**. The selectivity for CBP over BRD4 is highlighted, along with available PK data.

To build on this, the authors evaluated the binding mode of **7** in complex with CBP and BRD4(BD1) and found that the *C*2-substituent sits in a different conformation in each.^93^ They reasoned that constraining the molecule in the CBP binding conformation may lead to improved potency and selectivity. By introducing stereochemistry in the *N*1-linker, the (*S*)-enantiomer specifically (**CBP-30**), they were able to impart approximately 40-fold selectivity for CBP over BRD4(BD1) (*K*_D_ = 21 nM *vs.* 850 nM, respectively). **CBP-30** was also selective by thermal shift assay for CBP/p300 against other representative bromodomains, with only minor ΔT_*m*_ observed against the other BET proteins.^104^

In cells, **CBP-30** effectively displaced CBP from acetylated lysines at 100 nM, with no effect on BRD4 activity.^93^ It inhibited doxorubicin-induced p53 activity in a dose-dependent manner (IC_50_ = 1.54 μM), although it was unclear if this was due to CBP inhibition or its BRD4 inhibition at these concentrations. In addition, **CBP-30** was shown to inhibit IL-17A production at 2 μM in human cells derived from patients with ankylosing spondylitis and psoriatic arthritis, with a far more limited effect on gene expression observed than that associated with pan-BET inhibitor treatment.^104^**CBP-30** displayed moderate cytotoxicity (CC_50_ = 80 μM), which was above efficacious on-target concentrations. Despite good drug-likeness, *in vitro* ADME evaluation showed very rapid metabolism in human liver microsomes (HLMs), with no compound remaining after 60 minutes. These results precluded its use as an oral *in vivo* probe, but it remains a useful, selective *in vitro* probe for the function of CBP/p300.

#### CCS1477

**CCS1477** (Fig. 9) is a small molecule inhibitor of CBP/p300 developed by CellCentric for the treatment of castration-resistant prostate cancer, as well as AML and MM.^105–109^**CCS1477** bears the same 4-(1*H*-benzo[*d*]imidazol-5-yl)-3,5-dimethylisoxazole core as **CBP-30**,^93^ although with a reduced drug-likeness. A number of structures disclosed in the patent literature are structurally almost identical to **CBP-30**,^105^ and **CCS1477** is also the (*S*)-enantiomer, as observed with **CBP-30**.^93,110^

**CCS1477** has a *K*_D_ of 1.3/1.7 nM for CBP/p300, compared to a *K*_D_ of 222 nM for BRD4, with approximately 170-fold selectivity. It was selective over 32 representative bromodomains (<50% activity at 1 μM), as well as 97 kinases at 10 μM,^111^ showing no activity in a safety panel of 44 targets. **CCS1477** potently inhibited the growth of androgen receptor (AR) driven prostate cancer cell lines (IC_50_ = 49–230 nM),^112,113^ and induced significant growth inhibition in AML and MM cell lines at 100 nM.^114,115^ In mouse xenograft models of prostate cancer, AML, and MM, **CCS1477** induced tumour growth inhibition at 20 mg kg^−1^ that persisted after dose cessation. It was orally bioavailable and displayed reasonable PK properties in various species, predicting good human PK and informing a dosing regimen for clinical trials.

**CCS1477** entered the clinic in July 2018, in a phase I trial for castration resistant prostate cancer (NCT03568656).^116^ In August 2019 it began further evaluation in a phase I trial for haematological malignancies (NCT04068597) and is due to begin analysis and dissemination of findings for both by December 2021.^117^

#### DOT1L

Lysine methylation is a dynamic post-translational modification essential for the regulation of gene expression, along with various other cellular processes.^118–120^ Numerous lysine residues on histone proteins are substrates for a variety of lysine methyltransferases (KMTs), enzymes which transfer the methyl group of *S*-adenosylmethionine (SAM) highly specifically for the site and degree (mono-, di-, and tri-) of methylation.^120^ One such KMT is disruptor of telomeric silencing 1-like (DOT1L), thus far the only known KMT for histone 3 lysine 79 (H3K79) methylation.^121,122^

Di- (me2) and tri- (me3) methylation of H3K79 is associated with active gene transcription and euchromatin formation^121,123^ thought to be due to its ability to prevent the formation of other transcriptionally repressive lysine methylation marks.^124^ DOT1L activity has been implicated in a subset of aggressive leukaemias that involve a chromosomal translocation of the mixed-lineage leukaemia (MLL) gene on chromosome 11q23.^125,126^ Typically, the MLL gene encodes a KMT responsible for H3K4 methylation,^127,128^ which is lost during the translocation.^125,126^ The remaining MLL protein is fused to a variety of partners that then bind and recruit DOT1L.^129–131^ The recruitment of DOT1L and its transcriptionally activating methyltransferase activity leads to the increased expression of pro-leukaemogenic genes.^131–133^ Knockout of DOT1L has been shown to reduce the viability of MLL cell lines,^134,135^ suggesting that DOT1L inhibition may be a useful therapeutic strategy for the treatment of these leukaemias.

### The probes: **EPZ004777** and **SGC0946**

**Fig. 10:**
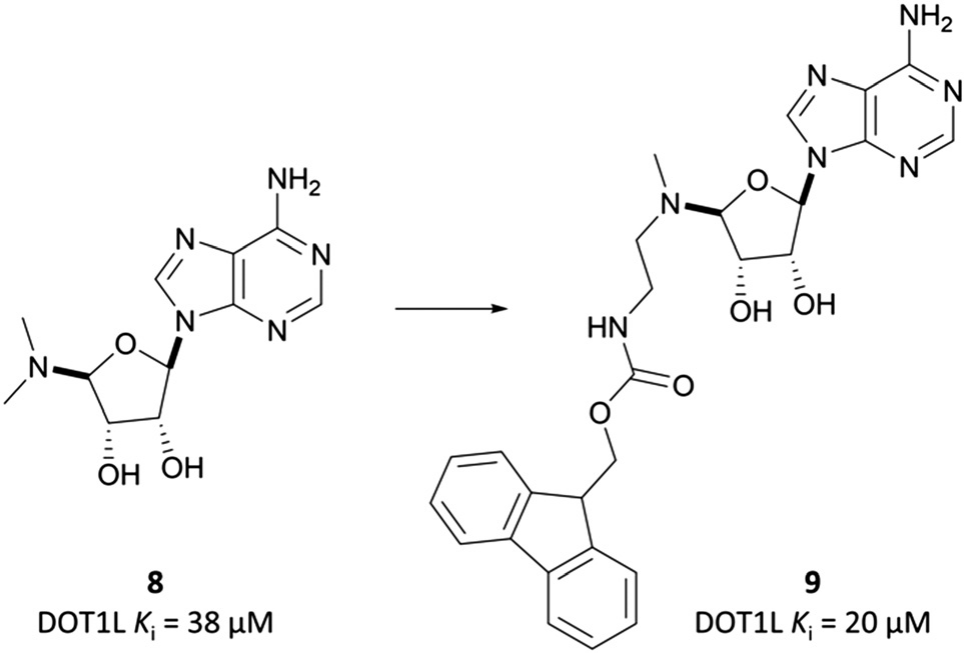
SAM-analogue **8** that was iteratively developed to the Fmoc-protected intermediate **9**.

Epizyme approached the development of its DOT1L inhibitor, **EPZ004777** (Fig. 12), through targeting the SAM cofactor binding domain.^136^ They began with a SAM analogue (Fig. 10, **8**), replacing the methionine moiety of SAM with a dimethylamino group. **8** bound in the same conformation as SAM with reasonable potency (*K*_*i*_ = 38 μM), validating its use as a starting point for derivatisation. They began to diversify the amine substituents in an attempt to extend into the lysine binding pocket of DOT1L and were surprised to find that an Fmoc-protected intermediate (Fig. 10, **9**) displayed modest potency (*K*_*i*_ = 20 μM).

**Fig. 11:**
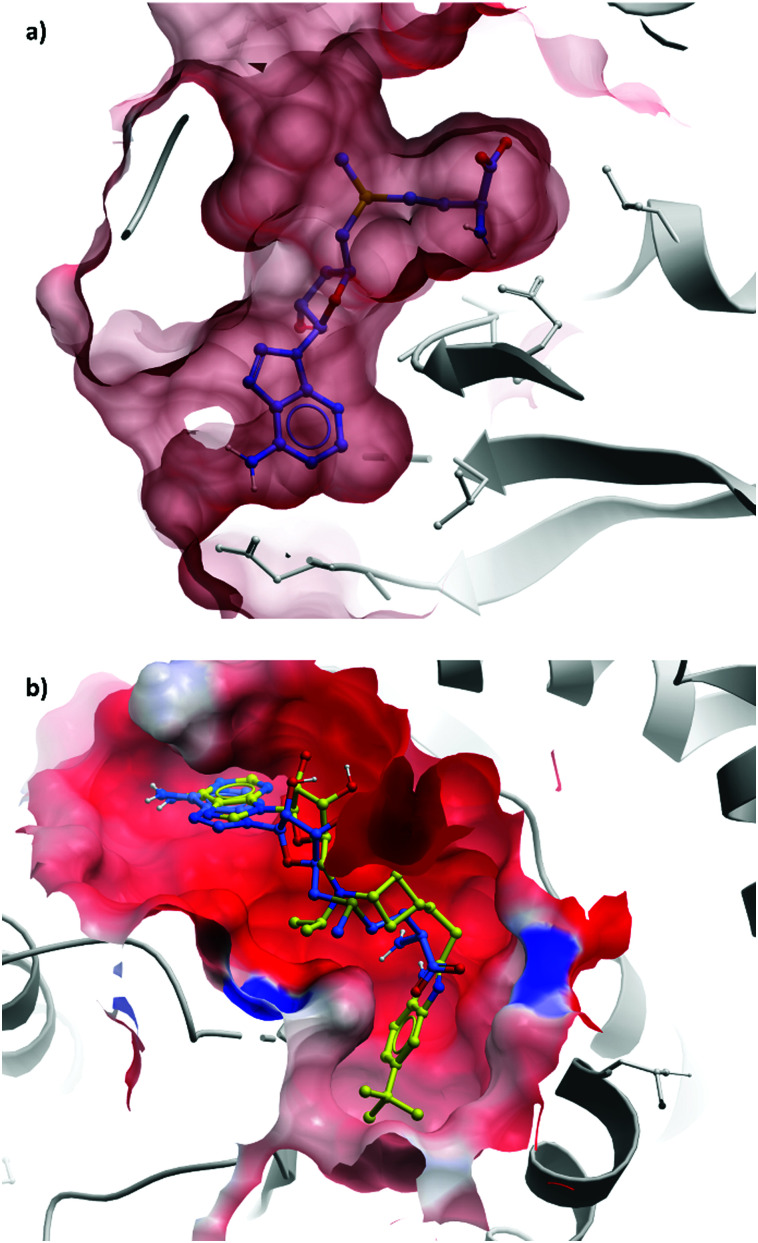
(a) Partially occluded view of SAM bound to DOT1L, showing the depth of the pocket occupied by the amino acid moiety (PDB: 3QOW); (b) overlaid crystal structures of SAM (blue) and **pinometostat** (yellow), showing the new hydrophobic cleft opened by the bulky *tert*-butyl benzimidazole group (PDB: 4HRA).

This finding led them to explore substituting the amine with a large hydrophobic group, linked by a short tether.^136^ Introduction of a *tert*-butyl phenyl urea and a propyl tether led to dramatic increases in potency. Modification of the nucleoside scaffold showed that deazapurine analogues were also potent inhibitors and combining the two led to **EPZ004777**, which had a *K*_*i*_ of 0.3 nM for DOT1L.^136^ The root of this potency was found to be the engagement of **EPZ004777** with a previously unknown binding pocket adjacent to the SAM-binding site, which wasn’t engaged by either SAM or its reaction product, *S*-adenosylhomocysteine (SAH). The urea of **EPZ004777** recapitulates the binding site interactions of the amino acid portion of SAM, whilst the bulky *tert*-butyl phenyl group induces a conformational change in a variety of hydrophobic residues, opening the pocket to accommodate it (Fig. 11b).^136^ As a result, and despite the universality of SAM as a cofactor, **EPZ004777** was highly selective for DOT1L over other KMTs, with >1000-fold selectivity.^129^

**Fig. 12:**
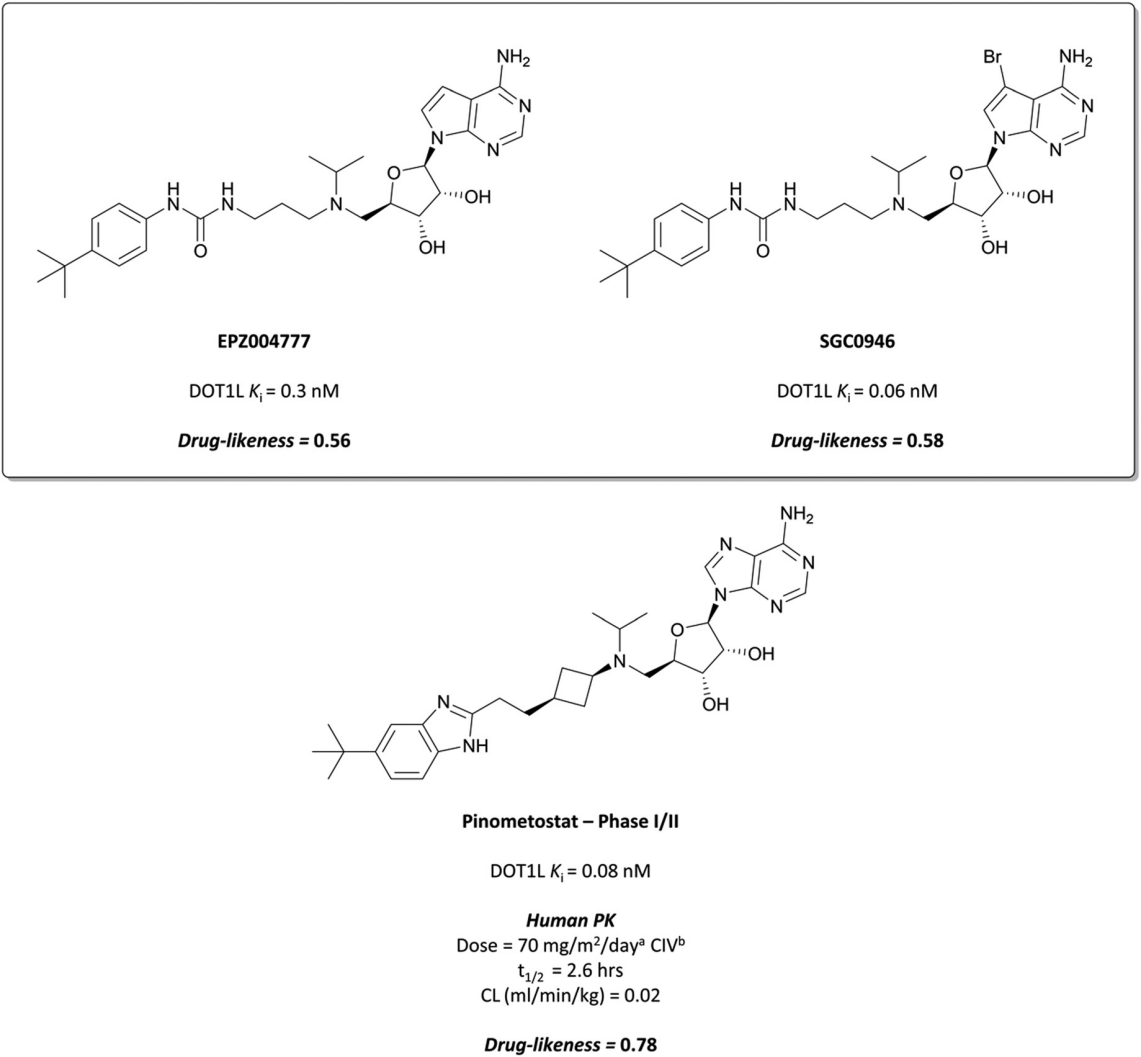
Probe **EPZ004777** and its brominated derivative with improved permeability, **SGC0946**. Clinical candidate **pinometostat** retains several structural features and pharmacophores from both molecules, as well as potency against DOT1L. ^*a*^ mg m^−2^ day^−1^ dosing based on body surface area. ^*b*^ CIV = continuous intravenous infusion.

Treatment of patient derived MLL-rearranged AML cells with **EPZ004777** resulted in a concentration-dependent, selective reduction in global H3K79me1 and H3K79me2 levels.^129^ It also reduced the expression of key MLL fusion target genes (IC_50_ (scintillation proximity assay (SPA)) = 700 nM), and induced apoptosis-mediated cell death in MLL-rearranged cells. *In vivo* testing in mouse xenograft models showed that H3K79me2 levels were reduced, efficacious doses were well-tolerated, and survival was extended.

**EPZ004777** displayed very poor permeability which precluded its clinical development,^129^ exemplified by the fact that it required administration *via* subcutaneous osmotic pumps to permit *in vivo* assessment. The scaffold was latterly improved following assessment of the binding pose of **EPZ004777** in the SAM binding pocket, which identified a hydrophobic cleft around *C*7 of the deazapurine ring.^137^ Bromination of this position yielded **SGC0946** (Fig. 12) and a five-fold increase in potency (*K*_*i*_ = 0.06 nM), along with an almost ten-fold increase in potency for abrogating H3K79me2 in MCF10A cells. **SGC0946** was also more active relative to **EPZ004777** in cells transformed with an MLL fusion oncogene, effectively reducing the expression of MLL target genes. **SGC0946** has subsequently been employed in a variety of settings to further elucidate the role of DOT1L in cancer and the mechanism of MLL fusion target gene expression.^138–141^

#### Pinometostat

The evident promise of DOT1L inhibition for the treatment of MLL-rearranged leukaemias led Epizyme to continue building on **EPZ004777**, eventually developing **EPZ-5676**, or **pinometostat** (Fig. 12).^142,143^ Reversion to an adenine scaffold over a deazapurine, the introduction of a cyclobutyl-based linker, and the recapitulation of the urea pharmacophore with a benzimidazole scaffold led to marked improvements over **EPZ004777**. With a *K*_*i*_ of 0.08 nM, **pinometostat** was nearly four-times more potent against DOT1L as **EPZ004777**, as well as displaying improved potency for inhibiting MLL-rearranged leukaemia cell proliferation, abrogating H3K79me2, and reducing the expression of MLL-fusion target genes. It also displayed improved selectivity, with greater than 37 000-fold selectivity observed over related protein methyltransferases, and displays improved drug-likeness as well.

Continuous intravenous infusion (CIV) at 70 mg kg^−1^ of **pinometostat** in a rat xenograft model of MLL-rearranged leukaemia caused complete tumour regression after 14 days with no observed weight loss or toxicity.^142^ This route of administration was necessary for maintaining plasma levels of **pinometostat** at efficacious concentrations, due to its poor oral bioavailability and short half-life. **Pinometostat** showed moderate to high clearance in mice, rats, and dogs, and was predicted to be a similarly moderate to high clearance compound in humans.

**Pinometostat** was progressed into the clinic for the treatment of adult and paediatric MLL-rearranged acute leukaemias.^144,145^ Interestingly, the observed clearance (0.08 L h^−1^) was far lower than predicted. This ‘vertical allometry’, as is observed with drugs like diazepam and warfarin,^146^ was found to be a result of binding to alpha1-acid glycoprotein (AAG), which is present in higher levels in human plasma than in mouse, rat, or dog plasma.^143^**Pinometostat** had a short half-life (2.6 h) and so was given by CIV over 28 days, displaying an acceptable safety profile however no clinical activity.^147^ Preclinical studies have suggested **pinometostat** may be more effective as a combination therapy,^148^ and a phase Ib/II study is currently ongoing to investigate the use of **pinometostat** in combination with the DNA-hypomethylating agent azacitidine^149^ as a treatment for MLL-rearranged leukaemias (NCT03701295, completed with results not yet reported).

#### G9a/GLP

In contrast to H3K79 methylation by DOT1L, H3K9 mono- and dimethylation by G9a and the closely related G9a like protein (GLP) is largely involved in the repression of transcription.^150–152^ The overexpression of G9a and GLP has been implicated in the progression of a variety of human cancers,^153–155^ along with addiction,^156^ neurodevelopmental disorders,^157^ viral infection,^158^ and peripheral neuropathy. Given the diverse range of cellular activities mediated by these methyltransferases, and the implications for disease treatment, a probe that could investigate the roles of G9a and GLP was highly sought after.^159^

### The probes: **UNC0638** and **A-366**

**Fig. 13:**
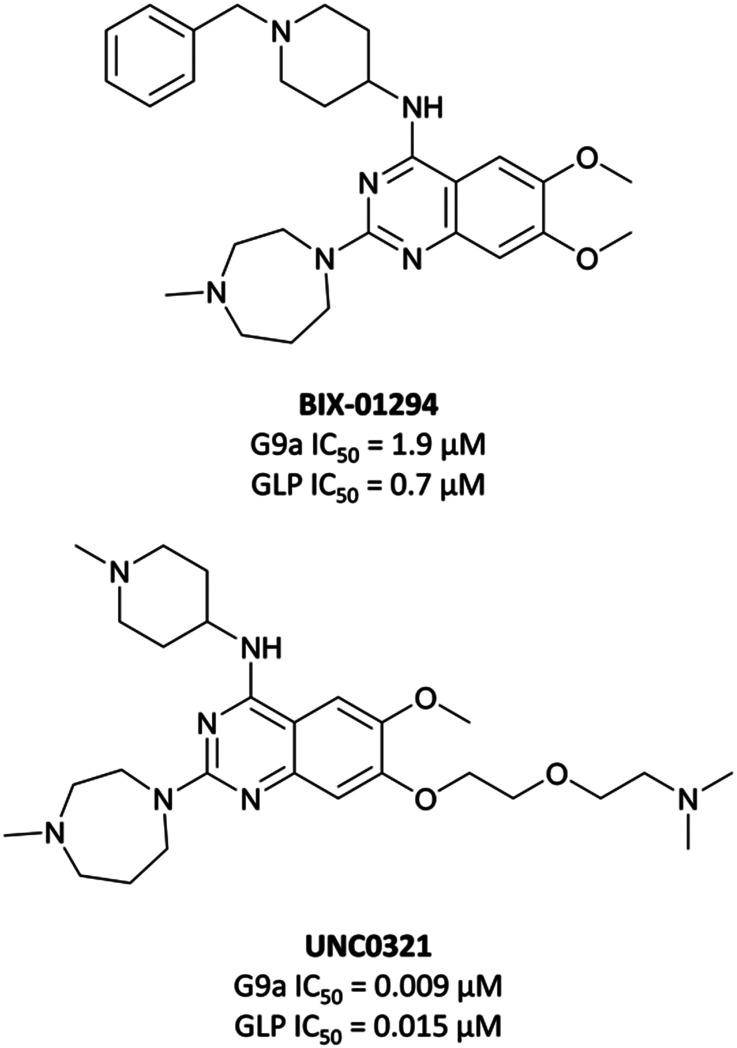
The first reported G9a/GLP inhibitor, **BIX-01294**, and the first iteration of more potent chemical probes developed following SAR, **UNC0321**.

The first reported inhibitor for G9a/GLP was **BIX-01294** (Fig. 13), a peptide-competitive inhibitor discovered after a high throughput screen (HTS) of compounds with potential KMT inhibitory activity.^160^ Whilst selective for G9a/GLP over other KMTs, it displayed only moderate potency (IC_50_ = 1.9 μM (G9a), 0.7 μM (GLP)).^161^**BIX-01294** reduced promoter-proximal H3K9me2 marks, although was found to only modestly increase transcriptional upregulation of G9a target genes.^160^ It has found use as a probe for the role G9a in cellular reprogramming^162,163^ and HIV-1 latency,^164^ however the concentrations required for cellular efficacy were cytotoxic.^165^

The lack of reported SAR around the scaffold of **BIX-01294** led researchers at the University of North Carolina at Chapel Hill, in collaboration with the SGC, to investigate the quinazoline template as a means of improving potency.^159,166–168^ The crystal structure of **BIX-01294** bound to GLP^161^ showed that the benzyl group on the piperidine ring lay outside the binding pocket, so its replacement should not lead to a loss in potency, whilst beneficially reducing MW and lipophilicity. It was postulated that modulation of the 7-methoxy moiety of **BIX-01294** would allow for penetration into the histone lysine binding channel of G9a/GLP, leading to further gains in potency. Diverse analogues of **BIX-01294** were synthesised to probe this SAR, resulting in **UNC0321** (Fig. 13), which had an IC_50_ (enzyme-coupled SAH detection (ECSD)) of 9 nM against G9a and 15 nM against GLP. The key structural difference between **BIX-01294** and **UNC0321** was the introduction of an ethoxyethyl-linked dimethylamino group that occupied the lysine binding channel of G9a/GLP. **UNC0321** was highly selective against other protein methyltransferases, however displayed reduced potency in cellular assays compared to **BIX-01294**, inhibiting H3K9me2 accumulation in MDA-MB-231 cells with an IC_50_ of 11 μM.^159^

**Fig. 14:**
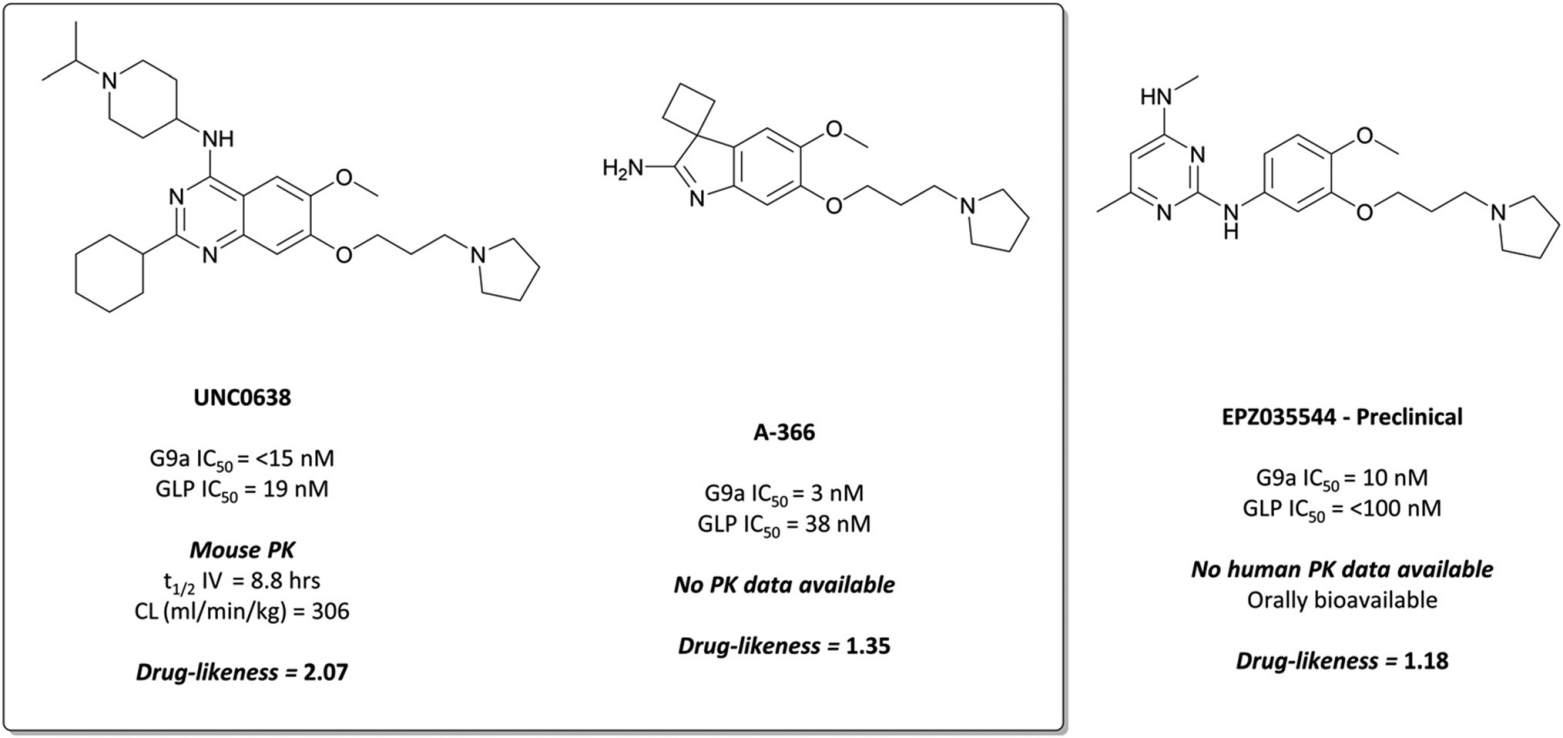
G9a/GLP chemical probe **UNC0638** and the structurally similar clinical candidate **EPZ035544**, which displays improved potency for G9a/GLP.

This lack of cellular potency was thought to be due to insufficient lipophilicity, leading to reduced membrane permeability.^159,168^ Increasing the lipophilic bulk of the 4-(piperidin-4-yl)amino capping group, substitution of the methylhomopiperazinyl group with a cyclohexyl group, and introducing a pyrrolidine in place of the dimethylamino group at the 7-position, yielded **UNC0638** (Fig. 14).^168^**UNC0638** was a potent G9a/GLP inhibitor (IC_50_ (ECSD) = <15 nM/19 nM), which reduced H3K9me2 levels in MDA-MB-231 cells with an IC_50_ of 81 nM, displaying vastly improved cellular potency compared to **UNC0321**. In all lines tested it reduced H3K9me2 by 60–80% at 250 nM, a comparable level to G9a/GLP knockdown.^159^**UNC0638** showed an improved toxicity/function ratio across several cell lines compared to **BIX-01294** at functional doses, as well as excellent selectivity against a range of epigenetic and non-epigenetic targets.^159^

In an effort to discover G9a inhibitors chemically distinct from the quinazoline based **BIX-01294** and **UNC0638**, researchers at the SGC Toronto in collaboration with Abbvie disclosed the development of **A-366** (Fig. 14).^169^ An initial spiro[cyclobutane-1,3′-indol]-2′-amine hit was combined with the tethered pyrrolidine of **UNC0638** to give **A-366**, which had an IC_50_ (SPA) of 3 nM against G9a and 38 nM against GLP. It was selective up to 50 μM against 20 HMTs and DNA (cytosine-5)-methyltransferase 1 (DNMT1), and reduced H3K9me2 levels in PC3 cells by 50% after dosing at 3 μM for 72 hours.^169^ It was also shown to induce modest (45%) tumour growth inhibition in a mouse xenograft model of AML, after 30 mg kg^−1^ dosing for two weeks.^170^

Despite primarily being used in investigations against cancer cells,^159,165,171,172^ G9a/GLP inhibition using **UNC0638** was also shown to induce the expression of foetal haemoglobin.^173,174^ In human red blood cells, it decreased repressive H3K9me2 levels and increased activating H3K9Ac levels at the locus of the foetal γ-globin gene. Inhibition of G9a also reduced recruitment of RNA polymerase II to the β-globin locus, leading to reduced expression of the β-globin gene. Together, this led to increased production of foetal haemoglobin and reduced production of the problematic β-globin chains of adult haemoglobin, suggesting inhibition of G9a may be of therapeutic benefit for haematological disorders such as sickle cell disease.

#### EPZ035544

These studies^173,174^ may well have inspired Epizyme to develop their own G9a/GLP inhibitor for the treatment of sickle cell, **EPZ035544** (Fig. 14),^175,176^ which shares similar structural features to **UNC0638** and **A-366**. Indeed, they cite the lack of *in vivo* data for **UNC0638** as inspiration for the development of **EPZ035544**.^176^ Drug-likeness remains consistently good across the compounds, highlighting the probes as useful templates for drug discovery.

**EPZ035544** is a potent inhibitor of G9a (IC_50_ = 10 nM), with a cellular IC_50_ of 55 nM against H3K9me2.^176^ It is highly selective against physiologically relevant targets (>2000-fold by *K*_*i*_ against KMTs, >1000-fold by *K*_*i*_ against kinases), and induces γ-globin expression in a dose dependent manner.^177^ The favourable PK properties and oral bioavailability of **EPZ03544** permitted extensive *in vivo* investigation of G9a inhibition in mice over 90 days. Doses of 50 and 75 mg kg^−1^ led to a 100-fold increase in the levels of embryonic haemoglobin mRNA observed in peripheral blood mononuclear cells (PBMCs), with quantitative mass spectrometry confirming concurrent increases at the protein level. The compound was well tolerated with no significant adverse effects reported in mice, leading Epizyme to advance **EPZ03544** further towards the clinic as a treatment for sickle cell anaemia. Their next iteration of compounds led to **EZM8266** (structure undisclosed);^178^ however preclinical toxicity concerns led to a discontinuation of the program.^179^

#### EZH2

H3K27me3 is a transcriptionally repressive mark mediated by the KMT polycomb repressive complex 2 (PRC2).^180–182^ PRC2 can be formed containing either of the KMT catalytic subunits enhancer of zeste homologue 1 (EZH1) or 2 (EZH2), leading to similar complexes that have markedly different repressive roles.^183–185^ PRC2-EZH2 catalyses H3K27me2/3 methylation, and its knockdown has been shown to affect global H3K27me2/3 levels, whereas PRC2-EZH1 has very little HMT activity.^184^ Rather, it acts directly to repress transcription by compacting chromatin.

Aberrant expression of EZH2 and hypertrimethylation of H3K27 have been implicated in a variety of cancers,^186^ including myeloma,^187^ lymphoma,^188,189^ prostate,^190,191^ and breast.^192,193^ As well as this, loss-of-function mutations of one of the lysine demethylases (KDM) responsible for demethylation of H3K27me3 (UTX) are associated with various renal and throat cancers, as well as with myeloma.^194^ Alongside its role in these cancers, PRC2-EZH2 is also integral in regulating cellular differentiation.^195^ As such, its evaluation as a safe and effective therapeutic target would require a chemical probe with favourable PK properties that would permit regular dosing to examine the effects of chronic inhibition.

### The probe: **EPZ005687**

**Fig. 15:**
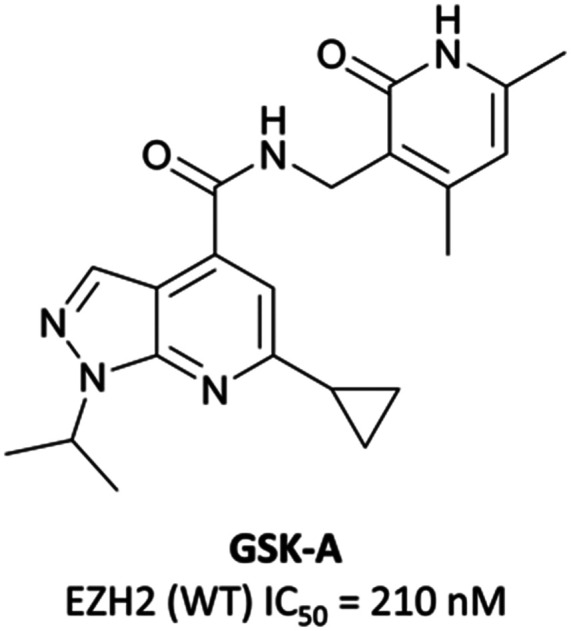
**GSK-A**.

To this end, groups at Epizyme and GSK disclosed almost concurrently (within ten days of each other) small-molecule inhibitors of EZH2 with similar structures, **EPZ005687** and **GSK126** (Fig. 17).^196–198^ Scientists at GSK had published details of an assay for the discovery of EZH2 inhibitors some months earlier, identifying **GSK-A** (Fig. 15) as a SAM competitive EZH2 inhibitor, with an IC_50_ (SPA) of 210 nM and a *K*_*i*_ of 700 nM.^199^ A breast cancer cell line was exposed to **GSK-A** for three days, resulting in a dose-dependent reduction in H3K27me3 levels (∼50% reduction at 8 μM) and identifying **GSK-A** as a cell permeable, specific EZH2 inhibitor.

An initial screen of 180 000 compounds carried out by Epizyme discovered a pyridone pharmacophore.^196^ Hit expansion led to a compound with a remarkably similar structure to **GSK-A** and an IC_50_ of 620 nM against EZH2. Replacement of a pyrazolopyridine with an indazole, the introduction of a 4-benzylmorpholine, and increased lipophilicity at the 1-position of the indazole eventually yielded **EPZ005687**, with improved solubility and potency over the initial hit. It inhibited EZH2 with an IC_50_ of 54 nM, acting as a SAM-competitive inhibitor, and was selective (>500-fold) against a variety of methyltransferases, with the exception of EZH1 (50-fold). It showed less than 50% inhibition against 73/77 GPCRs and ion channels at 10 μM, with the lowest IC_50_ for the remaining four 1.5 μM. **EPZ005687** remained potent against a variety of mutant enzymes associated with certain cancers,^200–202^ and also shows good drug-likeness.

**EPZ005687** displayed potent, selective reduction of H3K27me2/3 (IC_50_ = 80 nM) in cells^196^ and led to cell death in lymphoma lines bearing EZH2 Tyr641 and Ala677 mutations, with an IC_50_s of 300–400 nM across Tyr641 mutant cell lines. It was particularly potent against the Ala677Gly mutant line (IC_50_ = 36 nM), rendering it an incredibly valuable tool for the interrogation of the role of wild type (WT)- and mutant-EZH2 in the pathogenesis of disease.

#### GSK126

Building on the disclosure of **GSK-A**,^199^ GSK scientists began lead optimisation efforts.^197^ The pyridone group proved essential, whilst the pyrazolopyridine was replaced with an indole. Increasing lipophilic bulk of the *N*-substituent of the indole and introducing chirality improved potency, along with the introduction of a methyl group at the indole 3-position. Finally, replacement of the cyclopropyl with a 2-piperidylpyridine maintained good drug-likeness and resulted in **GSK126**.

**GSK126** displayed a similar *K*_*i*_ for both WT and mutant EZH2 (0.5–3 nM), a 1400-fold improvement in potency over **GSK-A**.^197^ It was greater than 1000-fold selective for EZH2 over 20 other human methyltransferases and displayed a selectivity of 150-fold over EZH1, despite a large degree of sequence homology. It induced a loss of H3K27me3 in mutant and WT EZH2 diffuse large B-cell lymphoma (DLBCL) lines at concentrations from 7–252 nM. Across a variety of lymphoma cell lines including Hodgkin's, non-Hodgkin's, and Burkitt lymphoma, DLBCL cells remained the most sensitive to EZH2 inhibition by **GSK126**, with seven out of 18 displaying sub-micromolar growth IC_50_s (28–861 nM) including both cytostatic and cytotoxic responses. A variety of transcriptional changes were observed, with chromatin immunoprecipitation sequencing (ChIP-seq) showing that the up-regulated genes were enriched for H3K27me3 prior to treatment, suggesting them as targets for EZH2.

These results also translated to mouse xenograft models of DLBCL.^197^ Complete tumour growth inhibition was observed at 50 mg kg^−1^, with regression observed at higher doses. Post-dosing at 50 mg kg^−1^, tumour stasis was observed, correlating with increased survival against vehicle-treated animals. As well as this, the compound was well tolerated, with no significant adverse effects reported.

**GSK126** entered a phase I trial in 2014 in patients with haematologic and solid tumours.^203^ There were adverse events reported in all patients, with one-third experiencing a serious adverse event. Eventually, dose-limiting toxicities and poor anticancer activity, along with a sub-optimal half-life, precluded further investigation of **GSK126** as a candidate for targeting EZH2 in patients.

#### Tazemetostat (**EPZ-6438**)

**Fig. 16:**
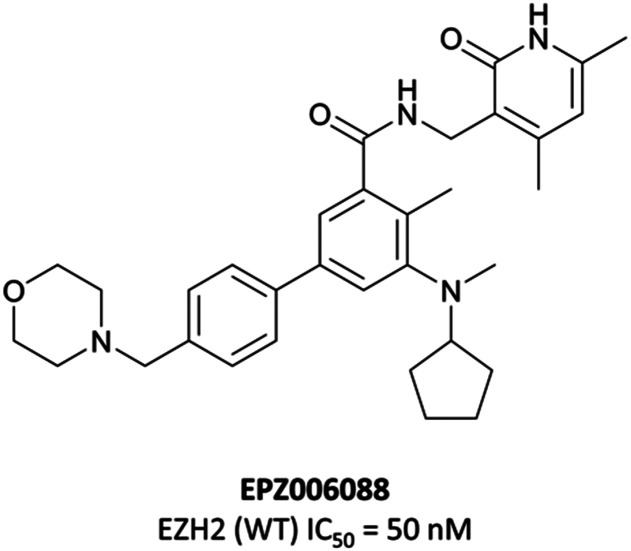
**EPZ006088**, a derivative of **EPZ005687** with a monocyclic core.

In parallele, Epizyme continued their development of an EZH2 inhibitor, building on **EPZ005687** which, despite good drug-likness, had suffered from poor bioavailability and high clearance.^204^ Modeling of its binding mode predicted that the indazole core of **EPZ005687** would force the pyridone moiety out of plane from the core to adopt an optimal binding pose (Fig. 19). This could be recapitulated by replacing the bicyclic system with a methyl-substituted aniline, with additional *N*-methylation further increasing potency. Opening of the five-membered ring also provided another vector along which polarity could be incorporated. A shift away from the bicyclic core initially resulted in **EPZ006088** (Fig. 16), with improved cellular H3K27me3 EC_50_ compared to **EPZ005687** (0.7 *vs.* 2.9 μM, respectively).

**Fig. 17:**
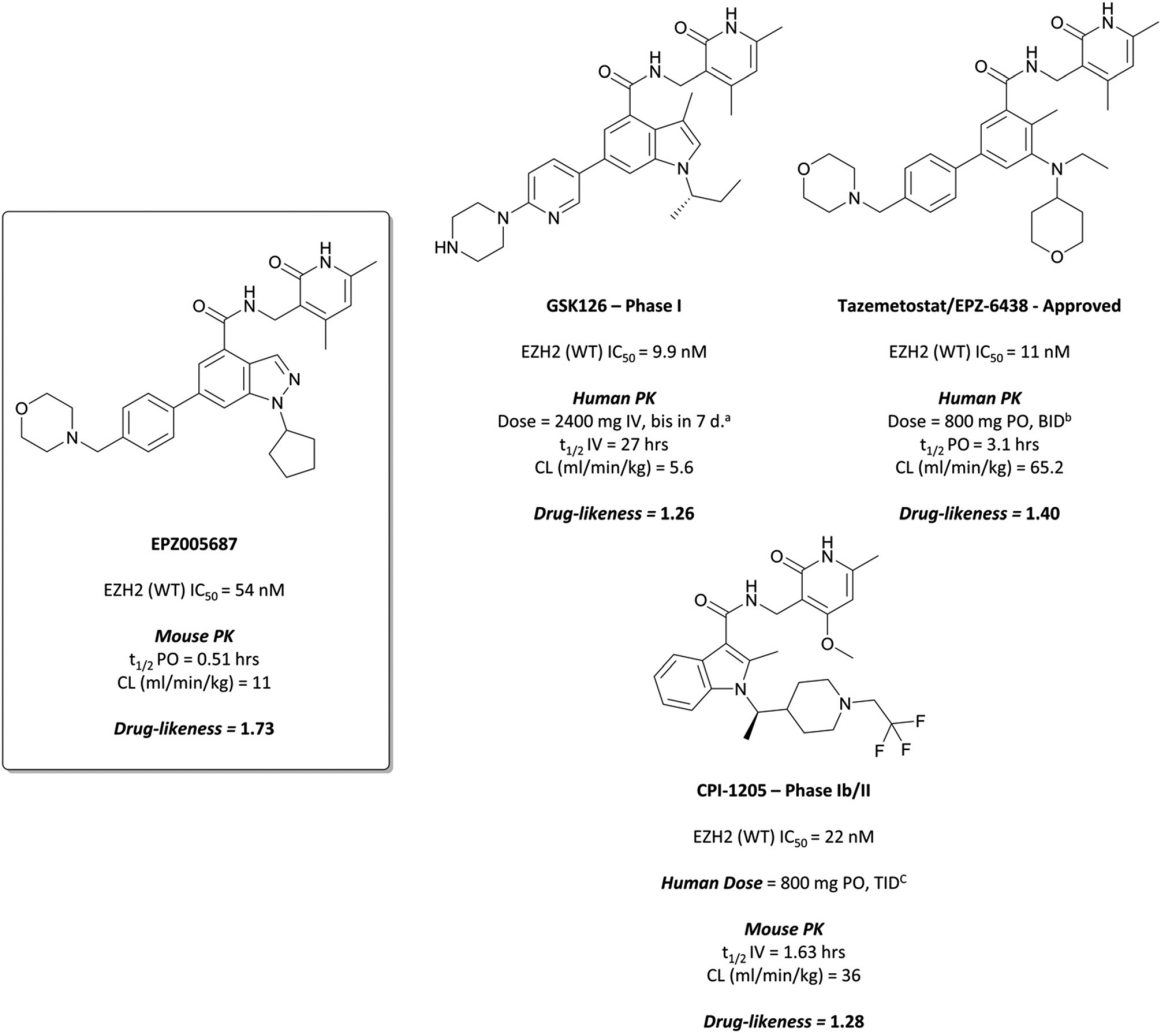
EZH2 chemical probe **EPZ005687**, containing the crucial pyridone and arylamide pharmacophores. The structurally related clinical molecules **GSK126** and **CPI-1205** are presented, along with the recently approved drug **Tazemetostat**. ^*a*^ Bis in 7 d. = twice weekly. ^*b *^BID = bis in die, twice daily. ^*c *^TID = ter in die, three times daily.

Further elaboration found that *N*-ethylation resulted in a modest increase in potency.^204^ Replacing the cyclopentane with a tetrahydropyran led to no improvement in potency but crucially lowered the log *D*, resulting in lower clearance. These modifications preserved drug-likeness and led to **EPZ-6438** (Fig. 17), otherwise known as **tazemetostat**, which had a *K*_*i*_ of 2.5 nM against EZH2 but importantly displayed higher cellular potency (EC_50_ = 0.2 μM) along with reduced clearance and good oral bioavailability. **Tazemetostat** remained selective against EZH1 (35-fold) and 14 other HMTs tested (>4500-fold).^205^

**Tazemetostat** showed promise in a number of preclinical models of various tumour types.^205,206^ 14-day, 1 μM treatment of *SMARCB1*-deleted malignant rhabdoid tumour (an aggressive childhood cancer)^207^ cells led to a reduction in H3K27me3 marks with concurrent growth inhibition. In a mouse xenograft model, tumours were almost entirely eliminated following twice-daily 250 mg kg^−1^ dosing, with no re-growth observed 32 days post-dose.^205^ In an EZH2-mutant lymphoma models, **tazemetostat** potently induced a reduction in H3K27me3 marks (IC_50_ = 9 nM),^206^ and in mouse xenografts, dose-dependent growth inhibition was observed along with complete and sustained tumour regression after 28 days. EZH2^A682G^ mutant cells were particularly sensitive, with a dose of 114 mg kg^−1^ leading to tumour eradication after 28 days.

The success of the preclinical studies prompted evaluation of **tazemetostat** in a number of clinical studies.^208–210^ A phase I trial in non-Hodgkin lymphoma patients showed a favourable safety profile for chronic dosing, as well as antitumour activity in patients with refractory B-cell non-Hodgkin lymphoma. It was subsequently advanced to phase II for patients with advanced solid tumours or with B-cell lymphomas.^211^ In patients with follicular lymphoma, it showed an objective response rate in 69% of patients with EZH2 mutant tumours and 35% with EZH2 WT tumours, suggesting its use as a treatment for follicular lymphoma. A phase II trial for advanced epithelioid sarcoma resulted in 15% of patients showing objective responses to treatment.^212^ As a result, it was approved by the FDA in January 2020 for the treatment of advanced epithelioid sarcoma^213^ and in June 2020 for the treatment of follicular lymphoma.^214^**Tazemetostat** is also currently being evaluated in a phase II trial for paediatric patients with relapsed or refractory tumours with EZH2, SMARCB1 or SMARCA4 mutations (NCT03213665, expected completion September 2024).

#### CPI-1205

**Fig. 18:**
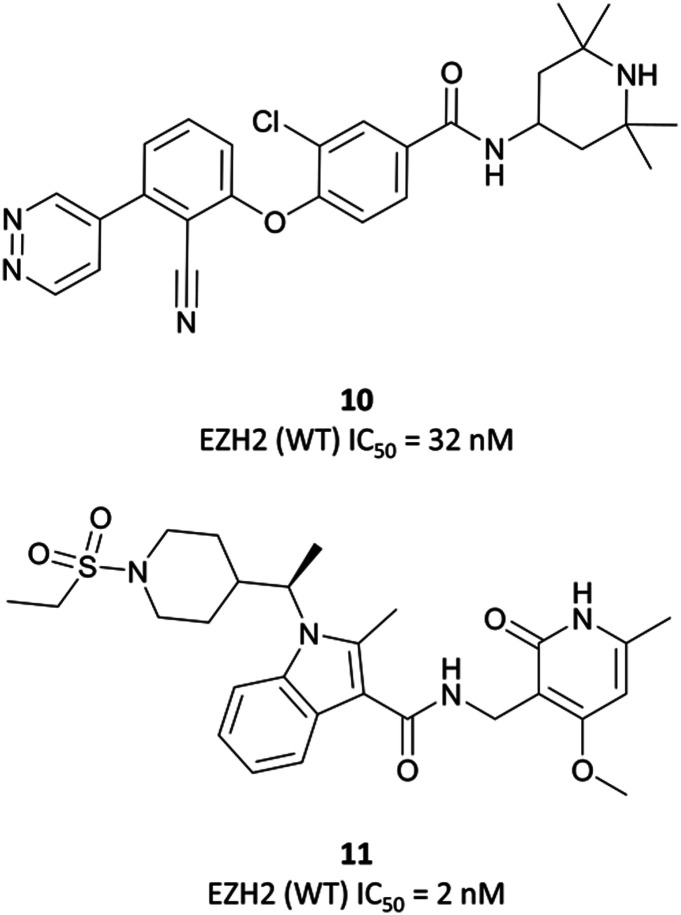
**10**, a non-pyridone-based EZH2 inhibitor. Poor cellular permeability led to the development of **11** following a second HTS.

Whilst researchers from Constellation Pharmaceuticals thought they had escaped the pull of the pyridone pharmacophore, they too eventually found it indispensable.^215,216^ They initially disclosed **10** (Fig. 18), a tetramethylpiperidinyl-based compound identified after iterative optimisation of a HTS hit against EZH2. **10** had an IC_50_ (SPA) of 32 nM against EZH2, and 213 nM against EZH1, however displayed a marked reduction in potency in a cellular context. It selectively reduced global H3K27me3, but with an EC_50_ = 7 μM, which the authors attributed to poor permeability.

**Fig. 19:**
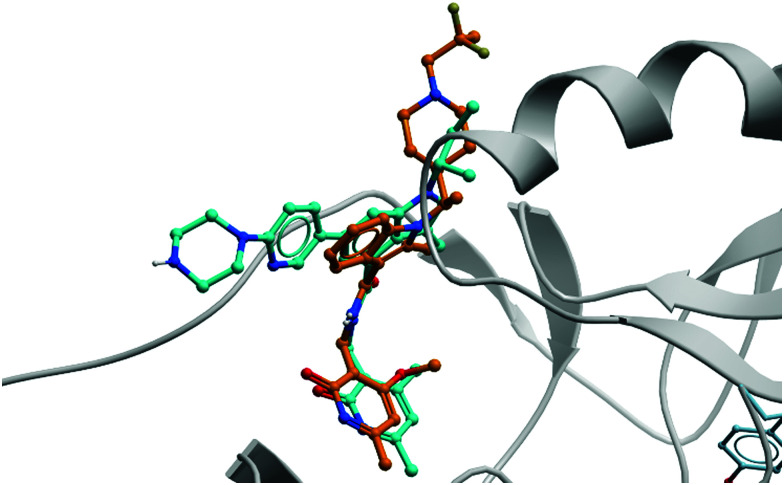
Crystal structure of **GSK126** (cyan) bound to EZH2 overlaid with the structure of a defluorinated analogue of **CPI-1205** (orange). The pyridone group is forced out of plane by the bicyclic core, occupying the same pocket in both structures (PDB: 5LS6 and 5WG6).

As the poor PK properties of **10** precluded its use *in vivo*,^217^ researchers revisited the initial HTS and identified a pyridone-based hit from which to begin optimisation studies. Attempts to replace the tetramethylpiperidinyl group in **10** with the pyridone proved fruitless, and so it was abandoned. Analogous to **GSK126**, they found that a central indole scaffold led to sub-100 nM potency, and as with **EPZ006088**, that forcing the amide bond out of plane with the central ring system was essential. This led to a series of *N*-substituted, 2-methyl indoles, the most potent of which was a 1-(ethylsulfonyl)piperidine derivative. A methoxy group was introduced on the pyridone scaffold to improve metabolic stability, yielding **11** (Fig. 18), which had an IC_50_ of 2 nM against EZH2 (WT) and an EC_50_ of 80 nM against H3K27me3 in cells. Subcutaneous dosing in a mouse xenograft model of lymphoma led to dose-dependent tumour growth inhibition, along with regression at higher doses.

Despite the improved profile of **11**, it was poorly bioavailable, and suffered from high clearance and a short half-life.^217,218^ Replacement of the sulfonamide with an *N*-trifluoroethyl group maintained potency and drug-likeness with improvements in oral bioavailability and half-life. The resultant compound, **CPI-1205** (Fig. 17), had an IC_50_ of 22 nM against EZH2 (WT) and a cellular EC_50_ of 32 nM against H3K27me3. In a mouse xenograft model of B-cell lymphoma, oral dosing at 160 mg kg^−1^ over 25 days led to significant tumour growth inhibition, with no significant weight loss observed. **CPI-1205** was selective against 30 other DNA and protein methyltransferases (>250-fold) and displayed no significant preclinical toxicity. As a result, it was advanced to the clinic in 2015 for the treatment of B-cell lymphoma.^219^ It was found to have an acceptable safety profile with some evidence of anti-tumour activity, leading to an expansion phase for lymphoma. It is also being investigated in phase Ib trials as part of a combination treatment for castration-resistant prostate cancer (NCT03480646, estimated completion date May 2021).^220,221^

## Type I PRMTs

Amongst the diverse protein posttranslational modifications, arginine methylation plays a key role in myriad cellular processes, including transcriptional regulation, cell signalling, mRNA translation, and cell-fate decision.^222–224^ Methylation does not affect the charge of the residue, but rather imparts greater bulkiness and hydrophobicity, which is important for its recognition by reader domains.^223,225^ The increased steric bulk as a result of methylation directs the position of the cation within the binding pocket, facilitating favourable cation-π interactions, and permitting discrimination between differing arginine methylation states (mono-methylation, symmetric dimethylation, and asymmetric dimethylation).

Type I protein arginine methyltransferases (PRMTs) are responsible for the mono- and asymmetric dimethylation of arginine residues, including PRMT1, -3, -4 (CARM1), -6, and -8.^222–224^ This is in contrast to type II PRMTs (PRMT5 and PRMT9) which catalyse mono- and symmetric dimethylation, and type III (PRMT7) which catalyse monomethylation. Type I PRMTs are implicated in a variety of human cancers:^226^ PRMT1 and PRMT6 dysregulation is associated with bladder and lung cancer,^227^ and PRMT4 has been found to be overexpressed in breast,^228^ colorectal,^229^ and prostate cancers.^229–231^

### The probes: **EPZ020411** and **MS-023**

**Fig. 20:**
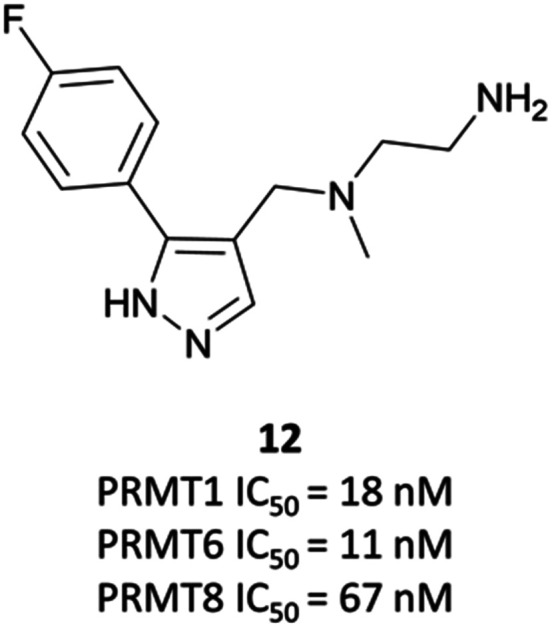
Type I PRMT inhibitor, and aryl pyrazole hit **12**.

The relevance of PRMT6 to human disease prompted Epizyme to pursue the development of a selective inhibitor for target validation studies.^232^ An initial HTS identified an aryl pyrazole hit (Fig. 20, **12**) as a potent inhibitor of PRMT1, PRMT6 and PRMT8. A crystal structure of **12** in complex with PRMT6 showed that the diamine motif occupied the arginine side-chain pocket, with the terminal amine engaging in favourable hydrogen bonding interactions with various residues and water molecules in the pocket. The pyrazole also engaged in hydrogen bonding, with the aryl ring forming favourable π–π interactions with aromatic residues. Coupled with general hydrophobic interactions, these contributed to the low nanomolar potency of the hit against the three PRMTs (IC_50_ (SPA) = 11 nM, 67 nM, and 18 nM against PRMT6, PRMT8, PRMT1 respectively).

**Fig. 21:**
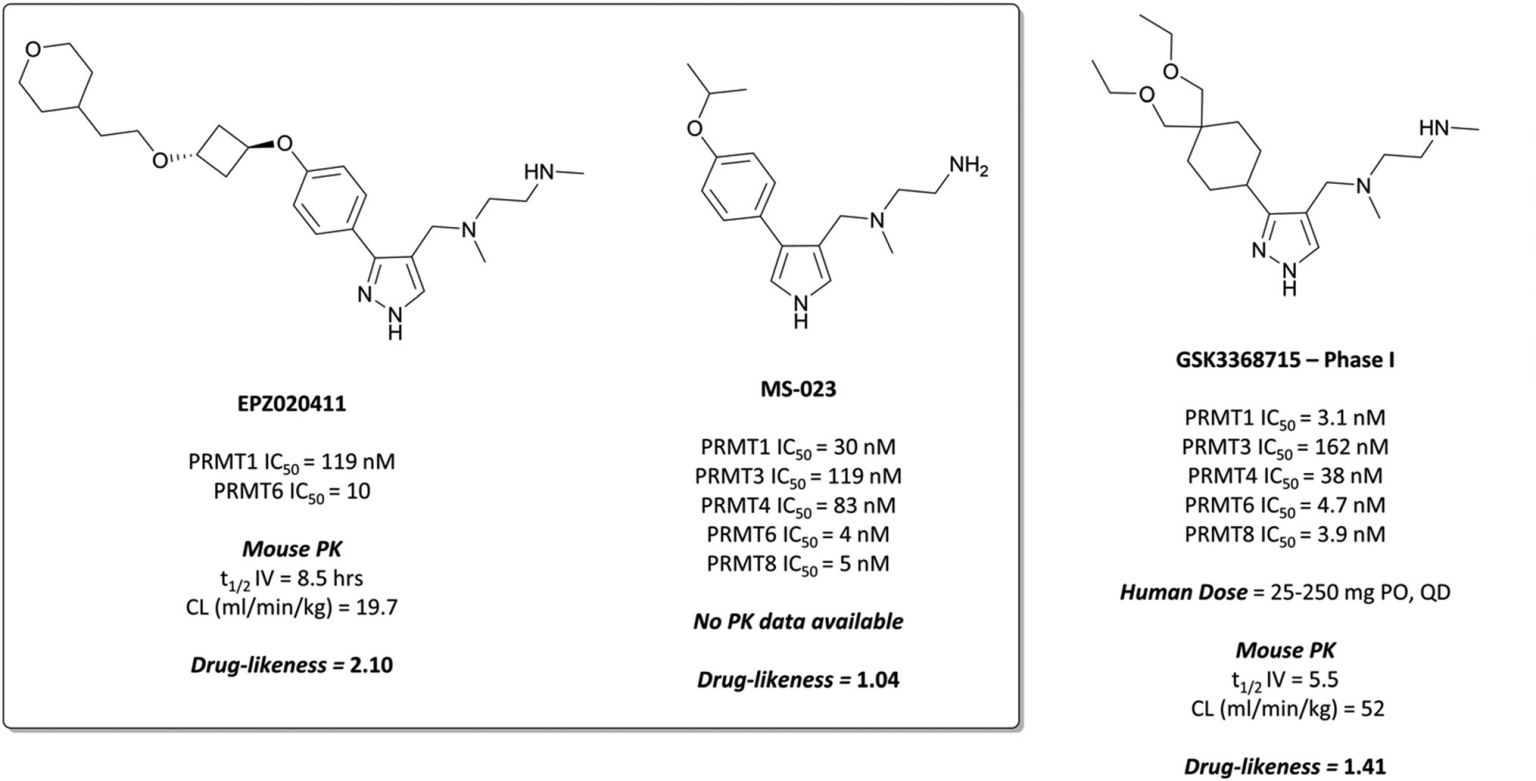
PRMT6 chemical probe **EPZ020411** and type I PRMT probe **MS-023**, alongside clinical candidate **GSK3368715**.

Regioisomers of the pyrazole were found to have no detrimental effect on binding,^232^ and selectivity for PRMT6 over PRMT1 and PRMT8 could be achieved through extension of the vector off the *para*-position of the aryl group. Introduction of an oxygen-linked alkyl group yielded **EPZ020411** (Fig. 21), with high drug-likeness and an IC_50_ of 10 nM against PRMT6 compared to 223 nM for PRMT8 and 119 nM for PRMT1. **EPZ020411** was 100-fold selective for PRMT6, -1, and -8 compared to other histone methyltransferases, including four other PRMTs. It induced a dose-dependent decrease in the levels of PRMT6-mediated H3R2 methylation (IC_50_ = 637 nM) and was found to be 10-fold less potent against a PRMT1-specific monomethyl arginine mark. PK evaluation showed moderate clearance and reasonable half-life, however poor permeability which translated to poor oral bioavailability (<5%). Subcutaneous dosing resulted in good bioavailability (65.6%), with the unbound concentration remaining above the observed PRMT6 IC_50_ for longer than 12 hours.^232^ It was thus recommended as useful tool compound for *in vivo* PRMT6 target validation studies.

Inspired by the discovery of **EPZ020411**, researchers at Mt Sinai and the SGC Toronto began their own investigations into the development of a type I PRMT selective chemical probe.^233^ They recognised the ethylenediamino moiety as an arginine mimetic and thus essential component of any potential probe, but also that the bulky aryl substituent of **EPZ020411** was crucial for PRMT6 selectivity, so opted for smaller substituents to enable targeting of other type I PRMTs.

Evaluation of 1,2,3-triazole- and pyrrole-based probes led to better understanding of the contribution of ring electronics to binding.^233^ The *para*-vector off the aryl ring remained important for activity against type I PRMTs, with *meta*-substituted aryl rings displaying far lower potency. Iterative compound design resulted in **MS-023** (Fig. 21), which contained a pyrrole core, *para*-isopropoxy group, and a terminal primary amine, in contrast to the terminal secondary amine of **EPZ020411**. **MS-023** was highly potent against the type I PRMTs, with IC_50_s ranging from 4 to 119 nM across them (Fig. 21), with relatively good drug-likeness.

**MS-023** showed no inhibition of type II/III PRMTs up to 10 μM, nor of 25 KMTs and DNA methyltransferases (DNMTs), or of three KDMs.^233^ It was shown to be non-competitive with either SAM or peptide substrates, thought to be because affinity for the peptide derives from interactions removed from the arginine binding pocket, and thus inhibition has no effect on binding. Crystal structure analysis demonstrated that the ethylenediamine moiety did indeed occupy the arginine binding pocket, with the terminal amine forming both direct and water-mediated hydrogen bonds with residues in the pocket. Much like the interactions observed with **EPZ020411**, the pyrrole engaged in hydrogen bonding and the aryl ring in π–π interactions with a binding site tyrosine residue.

Cell assays showed that **MS-023** could reduce both PRMT1- and PRMT6-dependent arginine methylation marks in a concentration dependent manner (IC_50_ = 9 nM and 56 nM for PRMT1, -6 respectively). When evaluated as a pan-type I PRMT inhibitor, a global decrease in arginine asymmetric dimethylation was observed, along with a concurrent increase in monomethylation, an effect consistent with PRMT1 knockout.^234^ Cell growth arrest was observed at concentrations as low as 100 nM over 10 days in a breast cancer line. These results cemented **EPZ020411** and **MS-023** as first-in-class probes for the investigation of type I PRMT biology.

#### GSK3368715

In collaboration with Epizyme, GSK developed their own type I PRMT inhibitor as a chemotherapeutic agent.^235^ A compound screen against PRMT1 was followed by lead optimisation focusing on potency and PK properties, resulting in **GSK3368715** (Fig. 21), a potent inhibitor of the entire family of type I PRMTs, with IC_50_s in the range of 3.1 to 162 nM (Fig. 21). The similar structural features to **EPZ020411** and **MS-023** result in similarly good drug-likeness.

**GSK3368715** was selective over the remaining PRMTs (>100-fold) and several other methyltransferases with less than 20% inhibition observed at 10 μM.^235^ Crystal structures of the compound in complex with PRMT1, along with kinetic studies, suggested a similar mode of binding to **EPZ020411** and **MS-023**. **GSK3368715** induced a global loss of asymmetric dimethylarginine (ADMA) in a panel of cancer cell lines, with an IC_50_ of 13.6 nM, and a concurrent increase in monomethyl- and symmetric dimethylarginine.

Anti-proliferative effects were observed across a variety of cell lines derived from both haematological and solid tumours.^235^ The most sensitive lines to type I PRMT inhibition were lymphoma and AML lines, with some subsets of NSCLC and pancreatic cancer lines also displaying sensitivity. Favourable PK properties, particularly oral bioavailability, allowed for further evaluation of these effects in mouse xenograft models of solid and haematological malignancies. Once-daily oral dosing at 150 and 300 mg kg^−1^ led to significant tumour growth inhibition in models of DLBCL, pancreatic adenocarcinoma, clear cell renal carcinoma and triple-negative breast cancer.

**Fig. 22:**
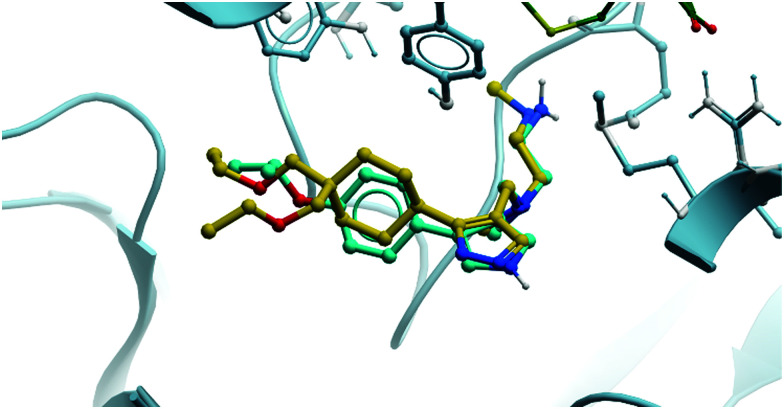
Comparison of the binding modes of **MS-023** (cyan) and **GSK3368715** (yellow), showing almost exactly the same pocket occupancy by both molecules.

As a result of the promising effect of pan-type I PRMT inhibition, and following further safety and PK profile evaluation, **GSK3368715** entered phase I trials for the treatment of solid tumours and DLBCL in 2018 (NCT03666988, expected completion date 2022).^235^ In the design of **MS-023**, it was noted that reducing bulk at the *para*-position of the aryl ring would enable the targeting of other type I PRMTs beyond PRMT6, a strategy that has yielded dividends. Indeed, overlaying the crystal structures of both compounds shows the same binding mode, occupying the pocket in almost exactly the same fashion (Fig. 22).

#### PRMT5

Of the type II PRMTs, PRMT5 predominates, responsible for the mono- and symmetric dimethylation of arginine residues.^224,236^ Its methylation products have been shown to both repress^237–239^ and promote^240^ gene expression, and it has a role in diverse cellular processes including tumorigenesis.^236,241^ Its overexpression has been observed in a variety of cancers, including lymphoma,^242,243^ lung,^244^ glioblastoma,^245,246^ breast,^247^ and colorectal.^248^ In addition, its transcriptionally repressive activity has been shown to impact several tumour suppressor genes.^237^ In addition to cancer, PRMT5 has been implicated in infectious disease, with both host and microbe PRMT5 playing a role in parasitic infections,^249^ Epstein-Barr virus,^250^ and some retroviruses.^251^

### The probes: **EPZ015666** and **EPZ015866**

**Fig. 23:**
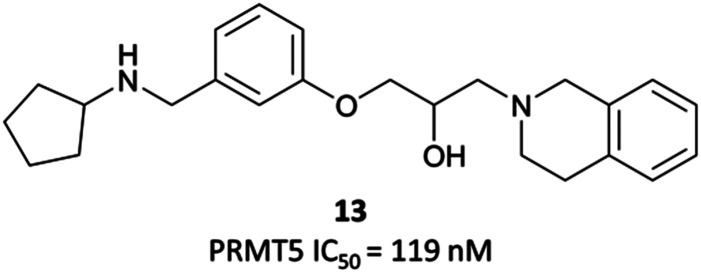
Tetrahydroisoquinoline hit **13**.

In order to validate its clinical relevance, researchers from Epizyme and GSK began development of a selective probe for PRMT5.^252,253^ An initial, peptide-competitive, tetrahydroisoquinoline (THIQ) hit (Fig. 23, **13**) from an HTS showed inhibition of PRMT5, with reasonable physiochemical properties. SAR around this scaffold demonstrated that the THIQ motif was essential, forming a cation-π interaction with the partial positive charge of the SAM methyl group. It was also involved in a π-stacking interaction with Phe327, and removal of the THIQ phenyl ring led to a complete loss of activity.

**Fig. 24:**
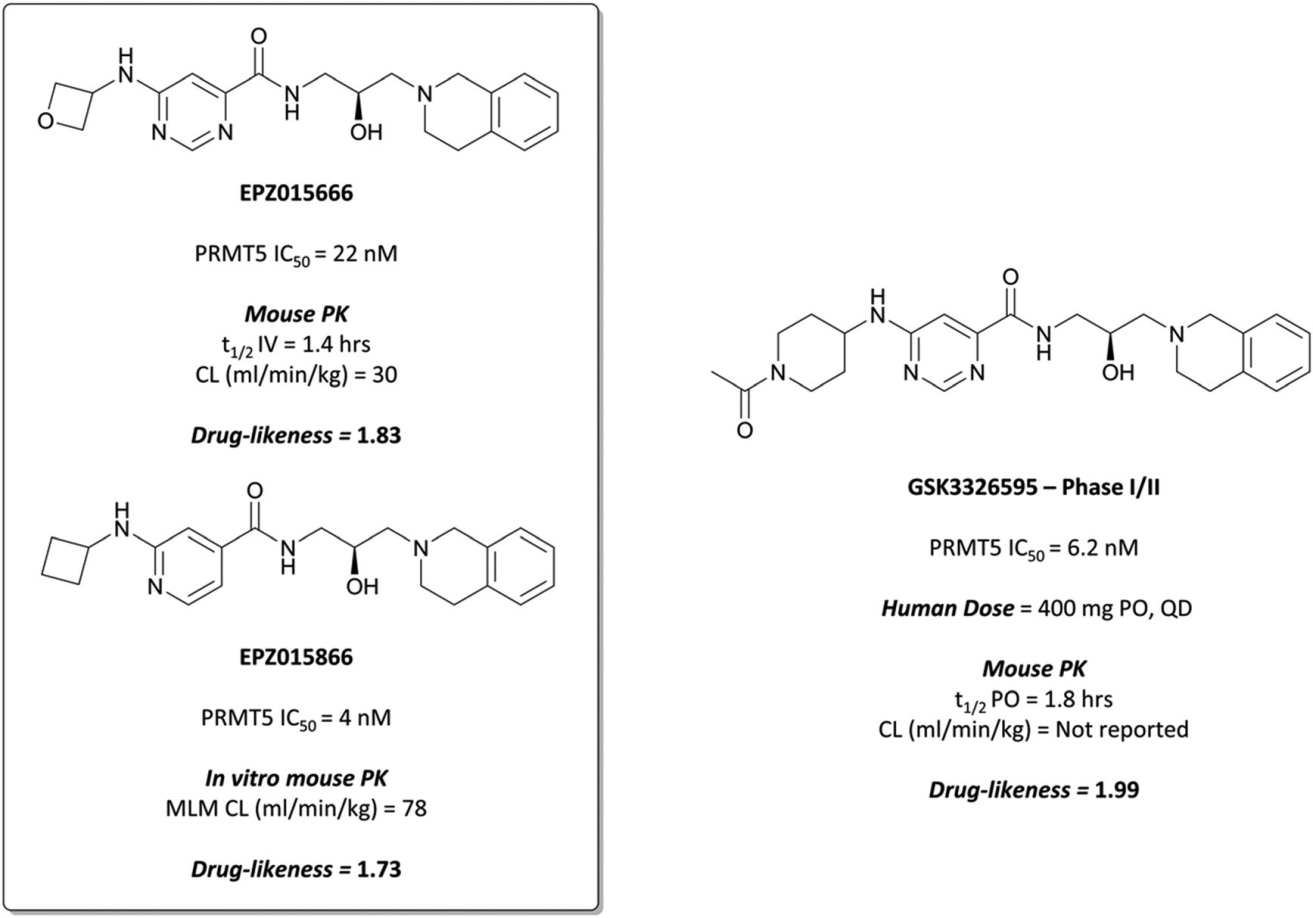
*In vivo* probe **EPZ015666** and *in vitro* probe **EPZ015866**, alongside the structurally derived clinical molecule **GSK3326595**.

Although their initial derivatives showed excellent potency, this was accompanied by significant instability in microsomal assays, with clearances approaching hepatic blood flow rates.^253^ To address this issue, they sought to reduce the clog *D* by incorporating less lipophilic amide analogues. In doing so, they identified **EPZ015666** (Fig. 24) and **EPZ015866** (Fig. 24), with IC_50_s of 22 nM and 4 nM respectively against PRMT5. Both retained potency but **EPZ015666** showed markedly improved clearance in both human and mouse liver microsomes. Both were highly selective over other protein methyltransferases, with no inhibition observed at 50 μM. **EPZ015666** showed a dose-dependent reduction in cellular symmetrical dimethylarginine marks, with IC_50_s (western blot) ranging between 4–347 nM in mantle cell lymphoma lines.^252^ Both **EPZ015666** and **EPZ015866** potently inhibited the proliferation of a lymphoma cell line, with IC_50_s of 351 nM and 62 nM respectively.

Despite good drug-likeness, the poor PK properties of **EPZ015866** precluded its use *in vivo*,^253^ but **EPZ015666** was evaluated against mouse xenograft models of mantle cell lymphoma.^252^ Twice-daily oral dosing led to dose-dependent tumour growth inhibition with no significant weight loss observed at the highest dose. Both compounds represented the first PRMT tool compounds, with **EPZ015666** orally bioavailable and suitable for *in vivo* studies.

#### **GSK3326595**/**EPZ015938**

Further medicinal chemistry efforts and lead optimisation led to **GSK3326595** (Fig. 24), which bound PRMT5 with an IC_50_ of 6.2 nM.^254^**GSK3326595** is a direct derivative of probes **EPZ015666** and **EPZ015866**: the essential THIQ motif remains, as well as the stereochemistry of the alcohol functionality, however the oxetane of **EPZ01566** has been substituted for an *N*-acetylated piperidine, improving drug-likeness.

**GSK3326595** induced a reduction in global symmetric dimethylarginine in various cancer cell lines, with EC_50_s ranging from 2.5–180 nM, and was selective (>4000-fold) over 20 other methyltransferases. It inhibited growth and induced cell death in various cancer lines with gIC_50_s ranging from 2.5 nM to over 10 μM, and breast, AML, and MM lines displaying the highest degree of sensitivity. Subsequently, it was evaluated in a mouse xenograft model of lymphoma, resulting in dose-dependent tumour growth inhibition and regression following 21 days oral dosing, with no significant weight loss observed.

A phase I trial to evaluate the effect of **GSK3326595** in patients with solid tumours and non-Hodgkin's lymphoma began in August 2016 (NCT02783300).^255,256^ Thus far, there has been a strong pharmacodynamic effect observed, accompanied by clinical activity in several tumour types, with an expected completion date of April 2025. A second phase I trial of **GSK3326595** began in October 2018 for patients with myelodysplastic syndrome and AML (NCT03614728, expected completion date March 2023).^257^

### The probe: **LLY-283**

**Fig. 25:**
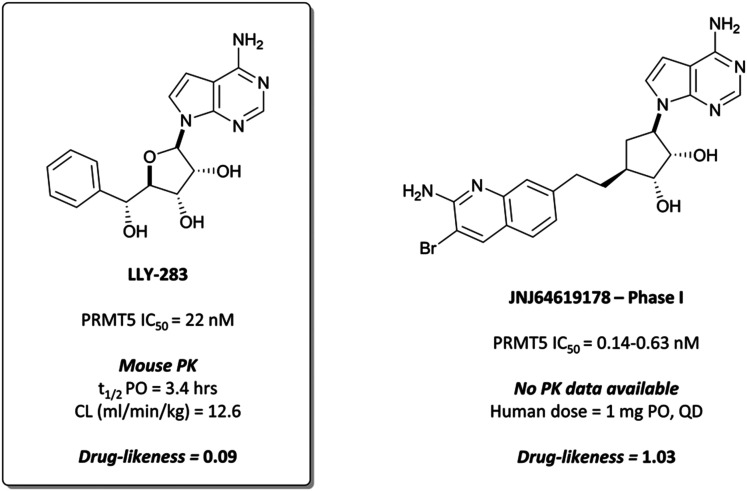
SAM competitive PRMT5 probe **LLY-283**, and the structurally related clinical candidate **JNJ64619178**.

In collaboration with Eli Lilly, researchers at SGC Toronto identified **LLY-283** (Fig. 25) as a potent, SAM-pocket binding inhibitor of PRMT5,^258^ in contrast to **EPZ015666** and associated peptide-competitive inhibitors. **LLY-283** is deazadenosine-based and adopts a similar binding pose in the pocket to SAM. The phenyl moiety causes a conformational change, acting to displace the Phe237 side chain which adopts an alternate structure compared to when bound to the peptide substrate.^259^

**LLY-283** was a potent inhibitor of PRMT5, with an IC_50_ (SPA) of 22 nM.^258^ Despite clear binding to the SAM pocket being observed, the compound wasn’t competitive with either SAM or the peptide substrate when evaluated against differing concentrations of each, with no clear explanation available. This had been previously observed for other compounds and methyltransferases (for example **MS-023**),^233^ however not when binding was occurring in the SAM pocket. **LLY-283** was selective against a panel of 32 other methyltransferases, with no loss of activity observed for any of the panel at 10 μM. It inhibited the symmetric dimethylation of SmBB′ protein with an IC_50_ of 25 nM in a breast cancer line and was effective at inducing antiproliferative effects in various cancer cell lines, with haematological tumours the most sensitive (IC_50_s (SPA) = 3–85 nM).

Favourable PK properties enabled oral dosing of **LLY-283** in a mouse xenograft model of melanoma. Once-daily, 20 mg kg^−1^ dosing over 28 days led to significant tumour growth inhibition, with no significant weight loss observed. Alongside the peptide-competitive inhibitors, **LLY-283** provides a useful addition to the toolbox of PRMT5 inhibitors.

#### JNJ64619178

The utility of SAM-inspired PRMT5 inhibitors was already apparent. A patent filed by Janssen Pharmaceuticals a year previously to the paper from the SGC Toronto^258^ described a series of compounds based on a similar carbanuceloside scaffold, with several displaying sub-nanomolar IC_50_s for PRMT5.^260^ At a 2017 meeting for the American Association for Cancer Research, they disclosed the discovery of **JNJ64619178** (Fig. 25) as a highly potent and selective inhibitor of PRMT5 with favourable PK and safety profiles.^261^ Whilst the structure has not been officially disclosed, it has been widely reported to be that in Fig. 25,^262–264^ which shows marked improvements in drug-likeness *versus***LLY-283**. According to the patent literature, the compound with the structure of **JNJ64619178** binds to the SAM-binding pocket with an IC_50_ of 0.63 nM,^260^ however it has been reported elsewhere that it has an IC_50_ of 0.14 nM.^263,265^ A diverse variety of cancer cell lines were sensitive to treatment with **JNJ64619178**, with target engagement confirmed by the inhibition of the symmetric arginine dimethylation of SMD1/3 proteins.^261,266,267^ In mouse xenograft models of NSCLC, small-cell lung cancer (SCLC), AML, and non-Hodgkin's lymphoma, tumour growth inhibition and regression were observed following once-daily oral dosing at 10 mg kg^−1^ and sustained post-dosing.

These results led Janssen to advance **JNJ64619178** to phase I trials in 2018 for the treatment of relapsed/refractory B cell non-Hodgkin lymphoma or advanced solid tumours (NCT03573310).^268^ Thus far, any observed adverse events have been manageable and there has been evidence of clinical activity. Full results are expected to be reported in July 2022.

## Beyond epigenetic probes

Whilst the focus of the review has thus far been on probes for epigenetic targets, there are other examples of chemical probes leading to drugs for other target classes.^7^ We shall examine a select few, along with the role they have played in inspiring clinical molecules.

## RIPK1

Receptor-interacting serine/threonine-protein kinase 1 (RIPK1) plays a key role in deciding the fate of the cell in response to various pro-death and inflammatory stimuli.^269^ It is involved in the regulation of necroptosis, a form of non-apoptotic cell death implicated in various inflammatory and neurodegenerative diseases such as multiple sclerosis (MS),^270^ amyotrophic lateral sclerosis (ALS),^271^ and Alzheimer's disease (AD).^272^ RIPK1 activity is regulated by tumour necrosis factor (TNF) binding to TNF receptor 1 (TNRF1), which leads to the RIPK1-kinase activity-dependent formation of a protein complex that induces necroptosis.^273–275^ Dependent on the context, RIPK1 activation can also cause apoptosis and neuroinflammation.^269^

### The probe: **GSK′481**

**Fig. 26:**
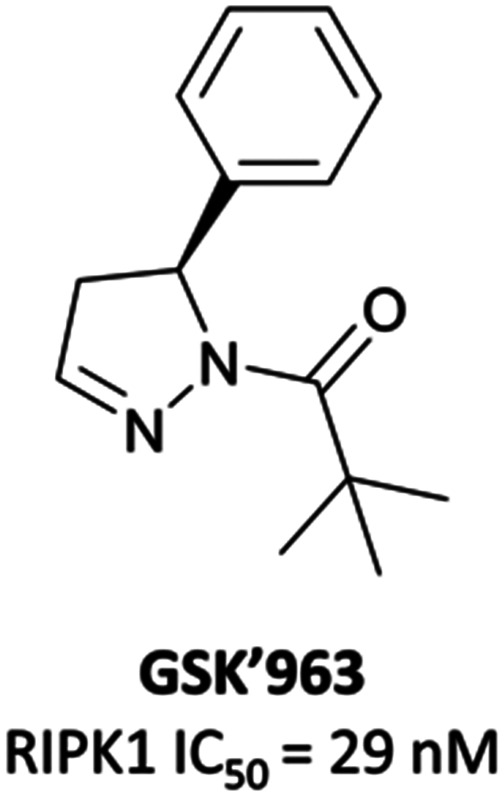
RIPK1 inhibitor **GSK′963**.

As a kinase, RIPK1 presents an attractive target for the development of small-molecule inhibitors of pro-necroptotic and pro-inflammatory pathways. The first identified RIPK1 inhibitors were the necrostatins, which displayed remarkable selectivity for RIPK1 over other kinases but suffered from relatively poor potency and metabolic stability.^276–278^ In an attempt to identify novel and improved RIPK1 inhibitors, scientists at GSK optimised an HTS hit and developed **GSK′963** (Fig. 26), which had an IC_50_ (FP) of 29 nM and was selective (>10 000-fold) for RIPK1 over 339 other kinases.^279^ Despite this, **GSK′963** had poor oral exposure in rodents, which precluded its development as an *in vivo* probe.

**Fig. 27:**
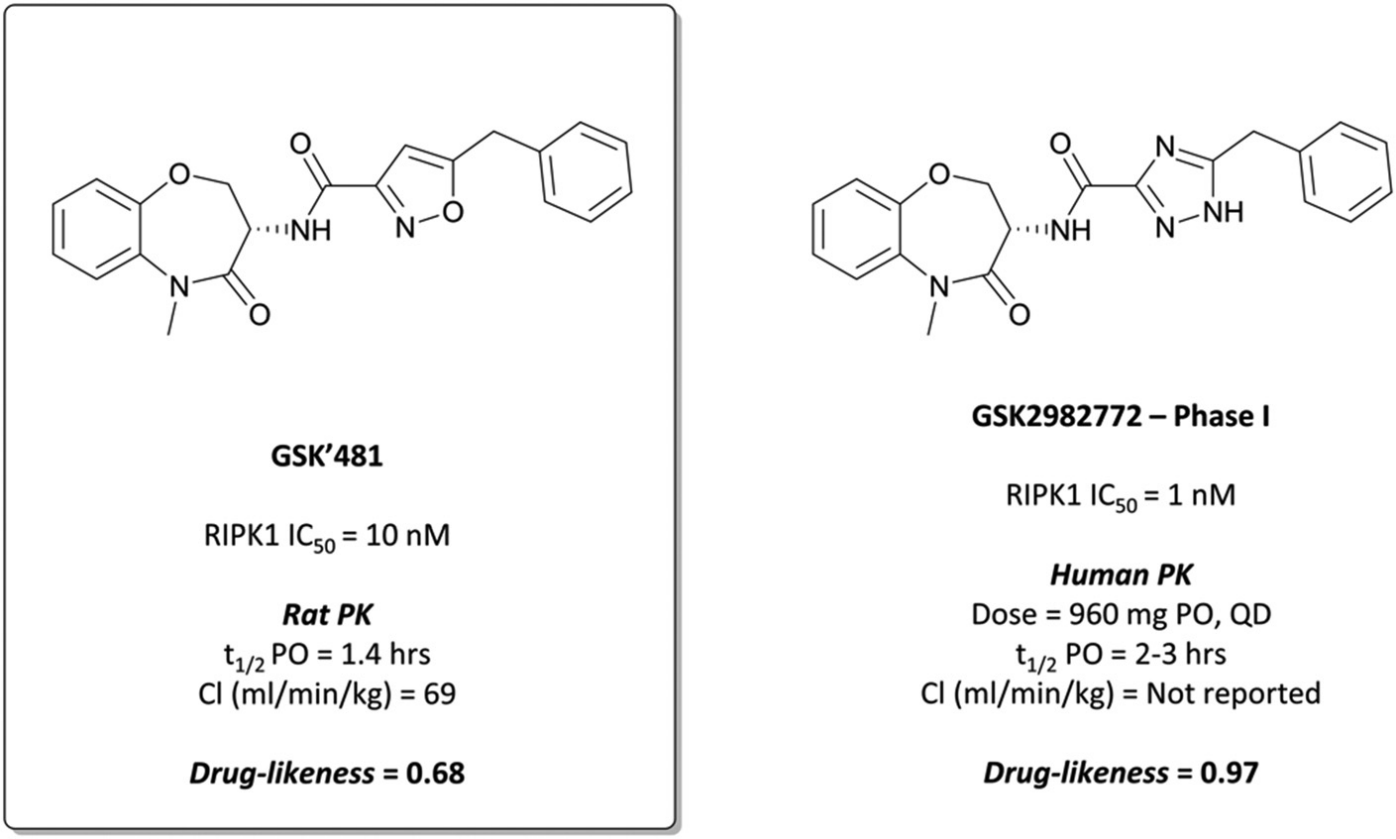
RIPK1 chemical probe **GSK′481**, from whose benzoxazepinone scaffold is derived clinical candidate **GSK2982772**.

Undeterred, the scientists at GSK screened a DNA-encoded small-molecule library against RIPK1 in the search for compounds that combined high potency and selectivity for RIPK1 with good PK properties.^280^ The screen identified a set of amino acid building blocks that were structurally distinct from more commonly known kinase hinge-binding motifs, combinations of which were synthesised and evaluated for potency against RIPK1. One such combination was the benzoxazepinone-based **GSK′481** (Fig. 27), which had an IC_50_ (FP) of 10 nM against RIPK1 and was selective (>7500-fold) over 456 other kinases.^280^ In the human monocytic U937 cellular assay, which measures the inhibition of necroptosis induced by TNF and a caspase inhibitor, **GSK′481** maintained its on-target potency with an IC_50_ of 10 nM. Despite this, a favourable PK profile remained elusive; **GSK′481** was very lipophilic with high clearance and poor oral exposure in rats.^281^ Nevertheless, its favourable *in vitro* profile makes it an attractive tool for probing RIPK1 biology in cells.

#### GSK2982772

**Fig. 28:**
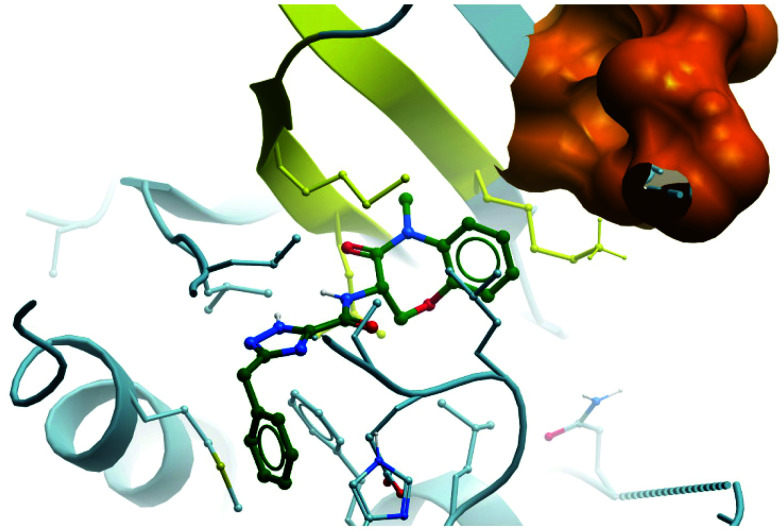
Binding mode of **GSK2982772** (green) to RIPK1, showing the tight pocket formed by two β-strands (yellow) around the benzoxazepinone moiety, with the ATP binding region shown in orange (PDB: 5TX5).

The key role of RIPK1 in TNF-mediated disease pathology motivated GSK to continue its pursuit of a small molecule inhibitor and eventual drug candidate.^281,282^ Building on **GSK′481**, they set about optimising its lipophilicity, solubility, and oral exposure.^281^ Due to the lack of a co-crystal structure, an homology model of RIPK1 was relied on to investigate SAR of the scaffold. Very little modification of the scaffold was tolerated, and any gains made in potency or one PK property were often at the expense of losses in others. For example, the introduction of an oxadiazolone on the benzo ring (**14**, Fig. 28) maintained potency and improved lipophilicity and oral exposure but had no impact on solubility.^281^

Eventually, modification of the central isoxazole led to the greatest improvements in lipophilicity, oral exposure, and solubility.^281^ The introduction of a 1,2,4-triazole resulted in **GSK2982772** (Fig. 27), which had an IC_50_ (FP) of 1 nM against RIPK1. Crucially, **GSK2982772** had a clog *D* of 3.8, which was a two-log improvement over **GSK′481** (clog *D* = 5.9), and displayed a seven-fold improvement in both solubility and oral exposure in rats, echoed in an improvement in drug-likeness. A crystal structure of **GSK2982772** in complex with RIPK1 showed it didn’t interact with any of the hinge residues and was instead binding more deeply within the ATP binding pocket (Fig. 28).^281^ The benzoxazepinone moiety sits in a pocket tightly flanked by two β-strands, explaining why any structural modification that resulted in a change in its conformation was not tolerated. This unexpected binding mode resulted in exquisite selectivity (>10 000-fold) for RIPK1 over 339 other kinases evaluated.

**GSK2982772** potently inhibited (as measured by the inhibition of RIPK1-dependent inflammatory cytokine production) necroptosis induced by caspase and apoptosis inhibitors in human primary neutrophils and whole blood (IC_50_ = 1.6 and 2 nM respectively).^281^ It reduced cytokine production from ulcerative colitis explant tissue in a dose-dependent fashion and was 93% protective in a mouse model of TNF-induced lethal shock after dosing at 50 mg kg^−1^. **GSK2982772** displayed favourable PK properties, including good clearance, half-life, and oral bioavailability.^281,283^

**GSK2982772** was advanced to the clinic for the treatment of peripheral autoimmune diseases in 2015.^283^ Excellent target engagement was achieved at well-tolerated doses, with no serious adverse events reported. Following this, it entered phase II trials in rheumatoid arthritis (NCT02858492), ulcerative colitis (NCT02903966), and plaque-type psoriasis (NCT02776033) in 2017.^283,284^ The drug was well tolerated however efficacy for these indications has not yet been demonstrated.^284,285^ It was re-entered into phase 1b trials in 2020 for psoriasis at a much higher dose (960 mg *vs.* 60 mg QD, NCT04316585), with results expected in October 2021.

## FXR

Bile acids are amphipathic molecules that act as regulators of a diverse array of processes, such as lipid and glucose metabolism.^286^ They undergo enterohepatic circulation which acts as an important means of regulating their synthesis and transport, along with exerting a wider effect on whole-body lipid metabolism. Bile acids are the natural ligands for the farnesoid X receptor (FXR), a nuclear receptor that plays a key role in this process.^287,288^ Activation of FXR initiates a cascade that reduces the conversion of cholesterol to bile acids by repressing the transcription of the cholesterol 7α-hydroxylase gene (CYP7A1), a critical enzyme for bile acid synthesis.^286,289^ As well as this, FXR regulates the expression of several lipid-modifying proteins, modulating lipogenesis through the reduction of triglyceride levels.^289,290^

Loss-of-function mutations in FXR and reduced FXR expression are associated with cholestasis, a condition resulting from the accumulation of bile salts due to impaired or obstructed bile flow.^291^ This build-up leads to the destruction of intrahepatic bile ducts and causes primary biliary cholangitis (PBC), a chronic and potentially fatal disease.^292^ FXR has also been implicated in nonalcoholic steatohepatitis (NASH), a condition caused by the accumulation of fat in the liver that leads to inflammation and fibrosis.^293^ As a result, FXR agonists that can suppress lipogenesis and the production of bile acids are of great interest as potential therapeutics for these diseases.

### The probe: **GW4064**

**Fig. 29:**
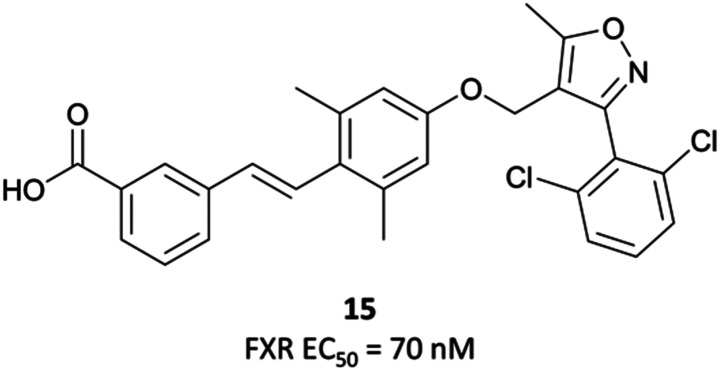
Initial isoxazole hit **15** from a combinatorial library screen against FXR.

Following the identification of FXR as a receptor for bile acids,^287,288^ scientists at GSK began a combinatorial library screen to discover a chemical tool to probe the pharmacology of the receptor.^294^ Initially, isoxazole **15** (Fig. 29) showed partial agonist activity (EC_50_ (FRET) = 70 nM) and was the basis for further exploration of SAR, with a focus on modulation of the three aromatic rings. Isoxazoles with bulky, lipophilic substituents were favoured, leading to the discovery of **GW4064** (Fig. 31), which had an EC_50_ (FRET) of 15 nM against the FXR. In cells transfected with human and mouse FXR expression vectors, **GW4064** had EC_50_s (FRET) of 90 and 80 nM respectively and was selective (>100-fold) against other nuclear receptors.^294^

**GW4064** induced a dose-dependent decrease in serum triglycerides in rats, with an ED_50_ of 20 mg kg^−1^, verifying a role for FXR in the regulation of lipid metabolism. **GW4064** had a modest half-life (3.5 hours) but was poorly orally bioavailable (10%), precluding further *in vivo* evaluation. Despite this, it was the first nonsteroidal FXR agonist identified and remains a useful tool for the probing of FXR biology.

#### Cilofexor (GS-9674)

**Fig. 30:**
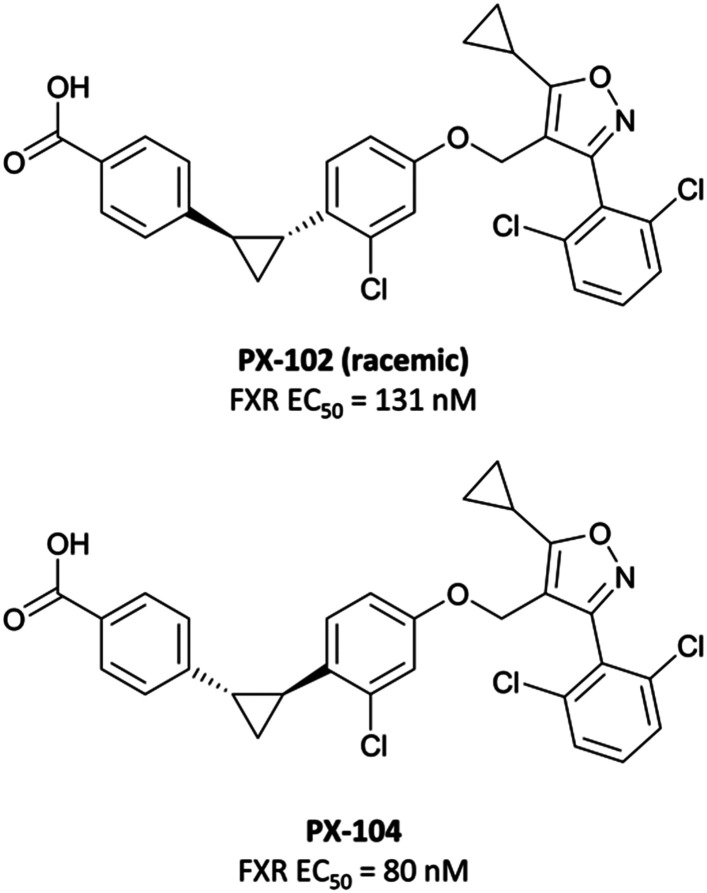
Racemic, cyclopropyl-based FXR agonist **PX-102** and its eutomer **PX-104**.

Since its disclosure in 2000, **GW4064** has pioneered a class of FXR agonists whose structures derive from the isoxazole scaffold.^295^ Alongside poor oral bioavailability, the stilbene moiety of **GW4064** was its undoing: a potentially toxic pharmacophore,^296,297^ it also conferred UV-instability making it unsuitable as a drug candidate.^298,299^ To improve on this, researchers from Phenex sought a linker that would match the conformational rigidity of the stilbene double-bond whilst alleviating the issues associated.^300^ Introducing a *trans*-cyclopropyl linker maintained potency and afforded PK properties that allowed for *in vivo* investigation of pharmacology. **PX-102** (Fig. 30) had an EC_50_ (FRET) of 131 nM against the FXR and had improved oral bioavailability in mice (44%) compared to **GW4064**.^294^**PX-102** showed potent activity against the FXR and induced transhepatic cholesterol efflux in both mice and monkeys,^301,302^ leading to its evaluation in phase I trials to assess safety, tolerability, and PK (NCT01998672, NCT01998659). The eutomer of **PX-102**, **PX-104** (Fig. 30), was progressed to phase II for the treatment of non-alcoholic fatty liver disease (NAFLD) (NC01999101).

**Fig. 31:**
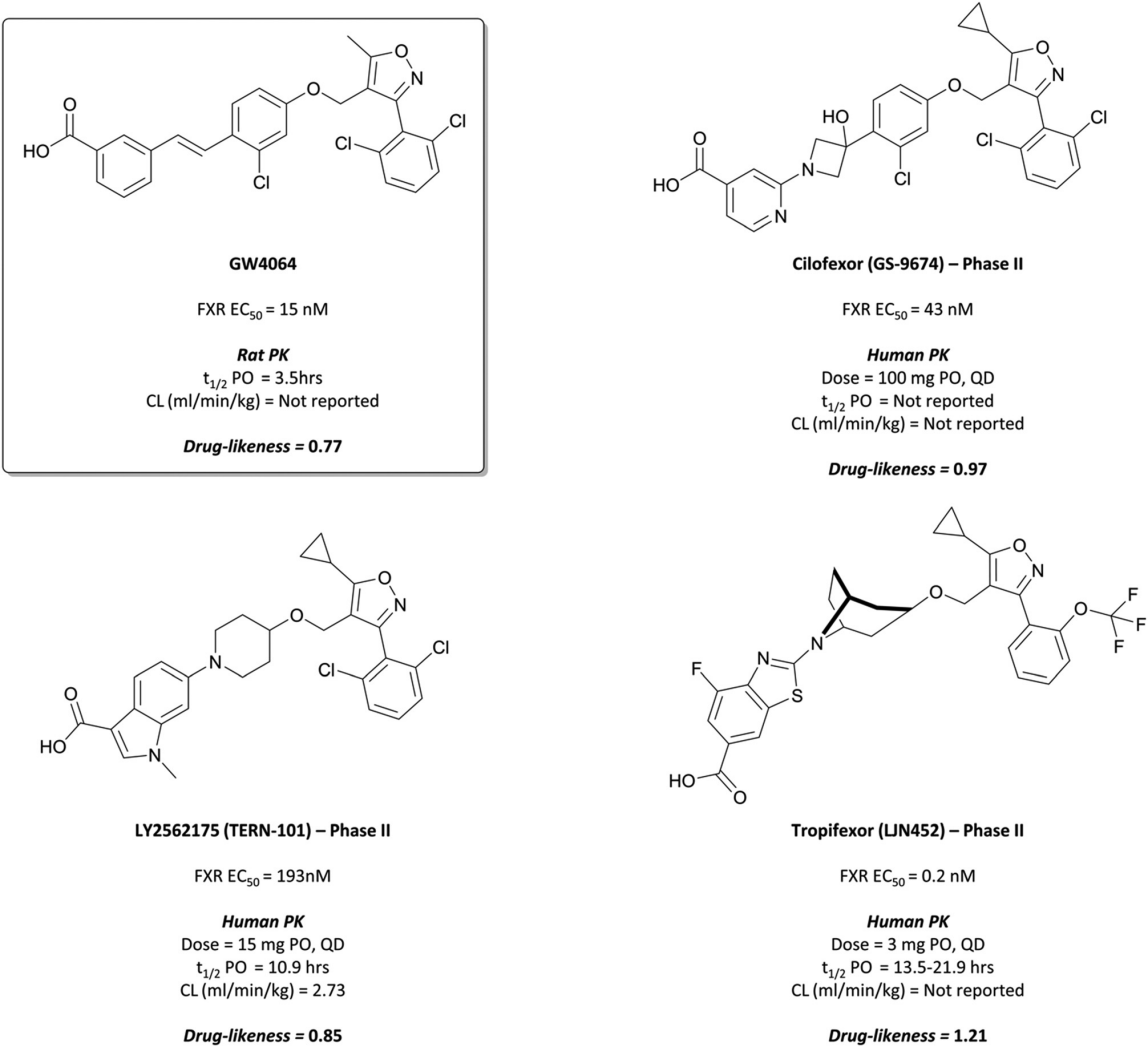
Pioneering early FXR agonist **GW4064** and the isoxazole-containing drugs it inspired: Cilofexor, LY2562175, and **Tropifexor**.

The results of these trials have not been published, however scientists at Phenex reported that the chiral cyclopropyl linker was synthetically inaccessible in a cost-efficient manner, leading to the need for appropriate achiral alternatives.^300^ This was achieved with the introduction of an hydroxy-azetidinyl linker, with further modification to include a 4-carboxylpyridine leading to the more drug-like **GS-9674**, or **cilofexor** (Fig. 31).^300,303^**Cilofexor** has an EC_50_ (FRET) of 43 nM for the FXR and demonstrated anti-inflammatory and anti-fibrotic effects in preclinical models of liver fibrosis.^304^ It is currently in phase III trials for primary sclerosing cholangitis (NCT03890120, expected completion August 2023). Thus far, it has shown significant reductions in bile acids and improvements in markers of cholestasis at tolerable doses, with no severe adverse events reported.^305^ As well as this, a phase 2 trial in patients with NASH (NCT02854605) demonstrated that at tolerable doses **cilofexor** induced significant reductions in hepatic steatosis (lipid retention) as well as serum bile acid levels.^306^

#### LY2562175 (TERN-101)

The demonstrated efficacy of FXR agonists in increasing levels of circulating high density lipoprotein (HDL) and reducing serum triglycerides^307^ prompted researchers at Eli Lilly to develop their own FXR agonist for the treatment of dyslipidaemia and atherosclerosis.^308^ Building on the isoxazole template of **GW4064**, they replaced the central phenyl ring with a piperidine and the terminal phenyl with an indole, improving drug-likeness and resulting in **LY2562175** (Fig. 31) which suffered from a loss in potency against FXR but possessed more favourable PK properties. **LY2562175** was only a partial agonist of FXR, with an EC_50_ (FRET) of 193 nM and 41% agonist efficacy when compared to **GW4064**, however it showed remarkable reductions in serum cholesterol and triglyceride in mice, with levels lowered by 80% and 76% respectively compared to vehicle treated animals after 10 mg kg^−1^ dosing.^308^

**LY2562175** was orally bioavailable and showed favourable PK properties across species (mouse, dog, and monkey), with low clearance and a good half-life.^308^ It was progressed to phase I for evaluation of safety and PK and was well-tolerated at doses as high as 600 mg, with a half-life of 16–24 hours supporting a once-daily dosing regimen. **LY2562175** was subsequently licensed to Terns Pharmaceuticals,^309^ where it was rebranded as **TERN-101** and evaluated in models of NASH^310^ (Terns poster ref). It was shown to reduce liver steatosis and inflammation, thus inspiring Terns to evaluate novel formulations of the drug in a phase I trial, investigating their absorption and PK^311^ (pharmacokinetics of two oral formulations poster). The tablet formulation gave faster absorption and better systemic exposure and is currently being investigated in phase II trials for the treatment of NASH (NCT04328077, results expected May 2021).

#### Tropifexor (LJN452)

**Fig. 32:**
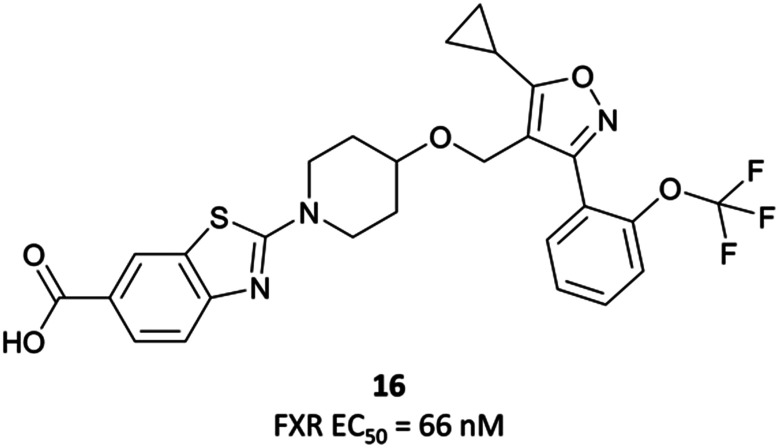
**LY2562175** analogue with improved potency and metabolic stability.

The discovery of **LY2562175** provided inspiration for Novartis to enter the FXR agonist space.^291^ Conversion of the indole of the partial FXR agonist to a benzothiazole improved the potency and led to full agonist activity. However, this compound suffered from high clearance in mice (50 ml min^−1^ kg^−1^) and very poor oral bioavailability (*F* = 6%), thought to be due to the metabolically susceptible dichlorophenyl moiety. To counter this, it was substituted for a 2-trifluoromethoxyphenyl group (compound **16**, Fig. 32), which had improved potency as well as clearance (24 ml min^−1^ kg^−1^) and bioavailability (*F* = 37%).^291^ Further improvements were gained by converting the piperidine linker to a nortropine scaffold, which was incredibly potent (EC_50_ (FRET) = 0.54 nM) however was poorly orally bioavailable (*F* = 11%) with a short half-life (1 hour). The simple introduction of a fluorine onto the benzothiazole solved this problem and yielded **LNJ452**, or **tropifexor** (Fig. 31), with the best drug-likeness of the series. It had an EC_50_ of 0.20 nM for FXR and in rats, low clearance (9 ml min^−1^ kg^−1^), along with improved half-life (3.7 hours) and bioavailability (*F* = 20%).^291^ It was also highly selective (>10 000-fold) for FXR over other enzymes, ion channels, nuclear receptors, and GPCRs.

In rats, **tropifexor** was shown to potently induce the expression of FXR-dependent genes at doses as low as 0.01 mg kg^−1^, and to lower the levels of serum triglyceride following oral administration.^291^**Tropifexor** displayed favourable PK properties across species, prompting its evaluation in a phase I trial for safety and tolerability. Doses up to 3 mg were well-tolerated, with a moderate rate of absorption and half-life of 13.5–21.9 hours, supporting a once-daily oral dosing regimen.^312^ It was subsequently entered into phase II trials for PBC, where it has been shown to reduce the amount of γ-glutamyl transferase (GGT), a liver enzyme that acts as a biomarker for liver disease(novartis reference).^313^ A once-daily dose of 0.09 mg for 28 days reduced GGT levels by 72% compared to a placebo, with no significant adverse effects reported. In a phase II trial for NASH, 0.2 mg **tropifexor** induced a 64% reduction of hepatic fat fraction with no serious adverse events reported.^314^

## BCL-2

Finally, an analysis of the value of high-quality chemical probes for drug discovery would be remiss without discussion of the development of the BCL-2 selective chemical probe **ABT-737** (Fig. 34),^315,316^ and how it was essential to the discovery of the highly effective chemotherapeutic **venetoclax** (Fig. 34).

The BCL-2 family of proteins are critical regulators of programmed cell death, functioning within a complex network of protein–protein interactions.^316,317^ The first identified, BCL-2 was found to inhibit apoptosis and prolong survival in cancer lines,^318,319^ and further anti-apoptotic members such as BCL-X_L_ and MCL1 were subsequently identified.^317,320,321^ These anti-apoptotic proteins are often highly expressed in cancer cells, contributing to tumour initiation and progression, as well as resistance to therapy.^322,323^ Targeting the BCL-2 family of proteins is challenging: extended, hydrophobic interactions with endogenous ligands^324^ and a high degree of sequence similarity with related proteins makes the design of selective and potent inhibitors incredibly difficult.^316^

### The probe: **ABT-737**

**Fig. 33:**
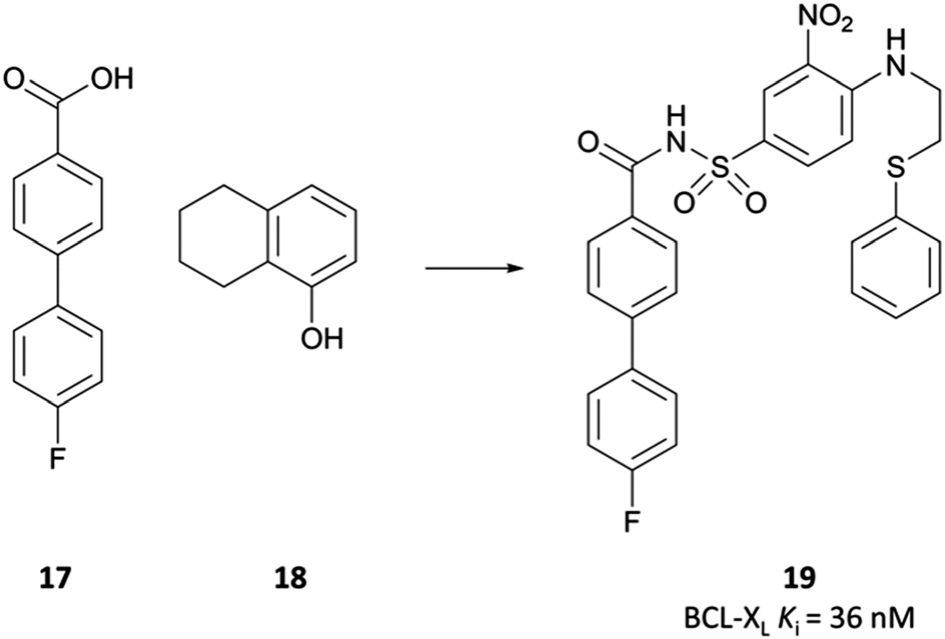
NMR screening identified compounds **17** and **18** that bound to distinct sites on BCL-XL. Linking the two, with some scaffold modulation, led to **19**.

In the pursuit of a potent inhibitor of the BCL-2 family of proteins, researchers from Abbott carried out a high-throughput NMR screen of a chemical library against BCL-X_L_.^315^ They identified two compounds (Fig. 33, **17** and **18**) that bound to two distinct sites in the BH_3_ binding cleft, observing similar binding modes to the endogenous peptide substrate.^324^ Substitution of the carboxyl of **17** with a bioisosteric acylsulfonamide provided an optimal vector through which to link the two sites, whilst maintaining the position of the acidic proton within the pocket. Further SAR led to compound **19** (Fig. 33), with **18** effectively replaced by a 3-nitro-4-(2-phenylthioethyl)aminophenyl group that spanned both initial binding sites. **19** had a *K*_*i*_ (FP) of 36 nM against BCL-X_L_ but showed markedly reduced activity in the presence of human serum albumin (HSA). Analysis of the crystal structures of **19** in complex with BCL-2 and HSA showed structural features that were solvent exposed in complex with BCL-2 were engaged in hydrophobic interactions in complex with HSA. To reduce affinity for HSA, these features were modified with polar groups, including the incorporation of a 2-dimethylethylamino group and a substituted piperazine. Finally, the introduction of a bis-phenyl moiety off the piperazine to probe a lipophilic pocket in the binding cleft resulted in **ABT-737**, which had a *K*_*i*_ (FP) of less than 1 nM for the highly homologous BCL-2, BCL-X_L_ and BCL-w, with activity maintained in the presence of HSA. **ABT-737** showed selectivity over the less homologous family members BCL-B, MCL-1, and A1 (*K*_*i*_ (FP) = 0.46 μM, >1 μM, >1 μM respectively).

**Fig. 34:**
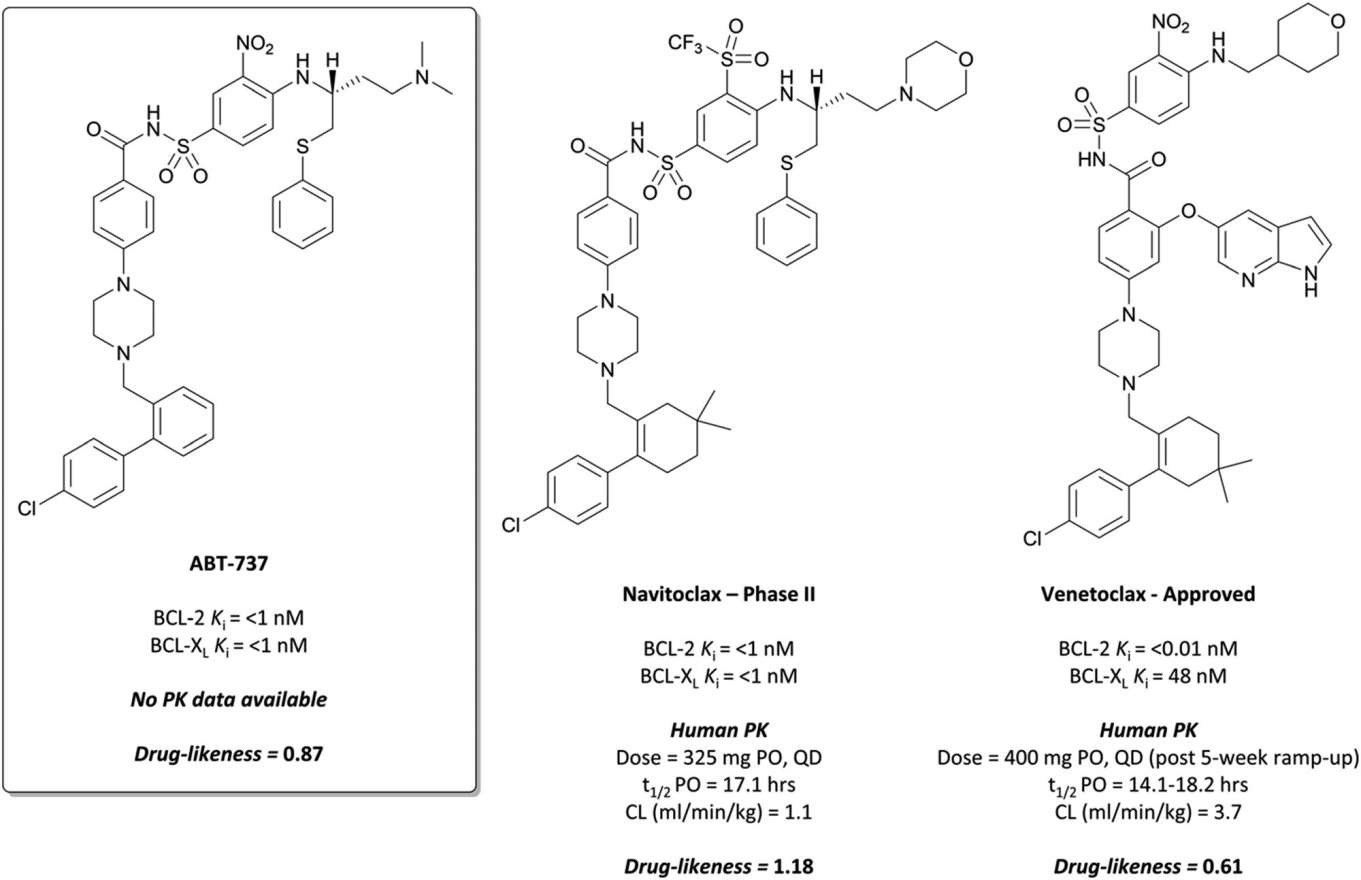
Chemical probe for the BCL-2 family of proteins **ABT-737**. Its derivative **Navitoclax** displayed DLTs in the clinic due to BCL-X_L_ inhibition. The next iteration, **Venetoclax**, Is selective for BCL-2 over BCL-X_L_, which led to far fewer adverse events and, coupled with high efficacy, its eventual approval for use.

**ABT-737** was shown to increase the sensitivity of various cancer cell lines to other chemotherapeutic agents, as well as to be cytotoxic itself, inducing cell death in patient-derived lymphoma cells at sub-micromolar concentrations.^315^ This cytotoxicity was replicated in solid tumours, with complete tumour regression observed in a mouse xenograft model of SCLC following intraperitoneal (IP) dosing at 100 mg kg^−1^. This was a breakthrough moment for investigating the BCL-2 family of proteins, an incredibly potent and selective probe that demonstrated excellent efficacy in cellular and animal models of disease.

#### Navitoclax (ABT-263)

Despite its evident promise as an anti-cancer agent, **ABT-737** suffered from poor oral bioavailability, limiting its potential for chronic dosing but confirming its status as excellent tool compound.^325^ This was perhaps unavoidable: the nature of inhibiting protein–protein interactions can imbue molecules with certain qualities undesirable in drugs when compared to the drug-like space defined by Lipinski: a molecular weight of less than 500, a clog *P* less than five, and fewer than five hydrogen bond donors and ten hydrogen bond acceptors.^79^ A quick glance at **ABT-737** shows it lies far outside this space, with both a high molecular weight and clog *P*. Nevertheless, the Abbott researchers decided to attempt to modify its scaffold in an effort to improve its physiochemical properties for oral exposure.

They began by exploring changes to the arylsulfonamide and amine functionalities, reasoning that modulation of their p*K*_a_s would alter the ionisation state of the molecule at physiological pH and improve oral absorption.^326,327^ They also investigated modifications to potential sites of metabolism, focusing on *N*-demethylation of the dimethylamino functionality and oxidative metabolism of the biphenyl moiety. Several initial changes that resulted in improved oral exposure also led to reduced cellular efficacy, however provided encouraging evidence that changes at these sites could result in the desired improvements.

Metabolism of the dimethylamino group was eventually mitigated with the introduction of a morpholine group, whose lower p*K*_a_ led to a modest improvement in oral exposure (4-fold) whilst maintaining cellular potency.^327^ Introduction of a triflone moiety to replace the aryl nitro substituent modestly increased the potency but crucially lowered the polar surface area, leading to a 7-fold increase in exposure. Finally, replacement of one of the phenyl groups on the lower half of the molecule with a *gem*-dimethylcyclohexene resulted in a sub-nanomolar cellular potency. Combining this with the above changes resulted in **navitoclax** (Fig. 34), which maintained its potency against BCL-X_L_, BCL-2 and BCL-w, but displayed a 22-fold higher oral exposure than **ABT-727** in mice, mirrored in an improvement in drug-likeness.

As a result, **navitoclax** was suitable for oral dosing in mouse xenograft models of SCLC and acute lymphocytic leukaemia (ALL).^325^ Once-daily, 100 mg kg^−1^ dosing led to complete tumour regression after 21 days, demonstrating equivalent effects to IP dosing of **ABT-737**. **Navitoclax** was subsequently advanced to the clinic for the treatment of chronic lymphocytic leukaemia (CLL), SCLC and other solid tumours.^328–330^ Some clinical activity was observed, however it induced thrombocytopenia in a dose-limiting fashion,^331^ and as a result it became difficult to safely reach effective concentrations in patients.

#### Venetoclax (ABT-199)

The decrease in the number of circulating platelets induced by BCL-2 family inhibition had previously been observed,^332,333^ and thought to be the result of direct BCL-X_L_ inhibition. The Abbott team had hoped these decreases would be well tolerated,^325^ going so far as to posit that circulating platelet levels could be a useful biomarker for BCL-X_L_ target engagement.^333^ This proved to be in vain, and the need for a BCL-2 selective compound became apparent.

They approached this by systematically removing key structural features to examine the effect on binding.^334^ It was discovered that removal of the thiophenyl motif imparted some selectivity for BCL-2, however with lower potency than **navitoclax**. A crystal structure of this analogue revealed that a second BCL-2 molecule had bound to form a dimer, with a tryptophan residue creating a π-stacking interaction with the trifloylaryl and a hydrogen bond with a binding site aspartate residue. This proved to be the key: this residue was one of the few not shared between the binding domains of BCL-2 and BCL-X_L_.

**Fig. 35:**
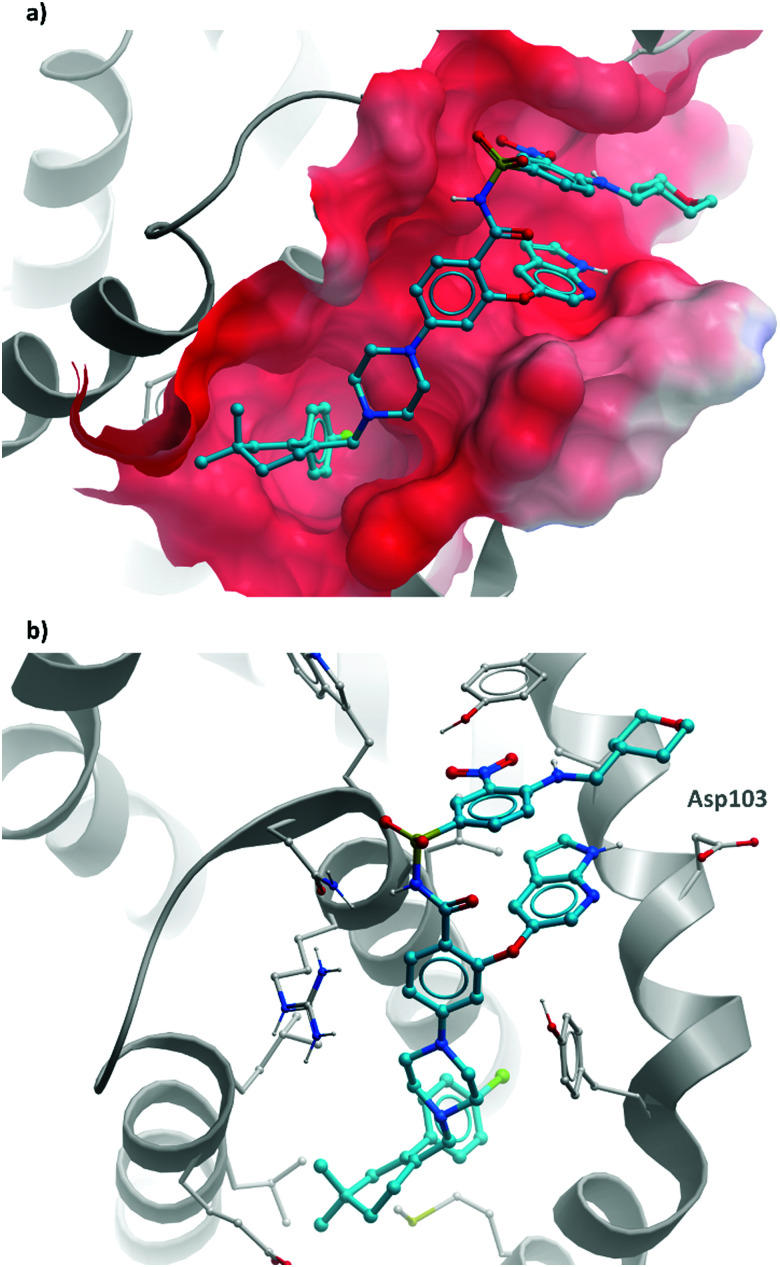
(a) Crystal structure of **Venetoclax** bound to the extended hydrophobic BCL-2 BH3 binding cleft; (b) the root of selectivity: Interaction of the azaindole moiety of **Venetoclax** with Asp103 in the BCL-2 binding site, which isn’t present in BCL-X_L_.

Introduction of an azaindole moiety tethered to the central core of the compound recapitulated the interaction, engaging the aspartate along with an arginine residue (Fig. 35b).^334,335^ Other subtle changes, for example substitution of the morpholine with a pyran, reintroduction of the nitroaryl, and a *gem*-dimethycyclohexene regioisomer resulted in **venetoclax**, which had a *K*_*i*_ (FP) of <0.01 nM for BCL-2 compared to 48 nM for BCL-X_L_ and 245 nM for BCL-w, greater than 300-fold selectivity. This selectivity was also observed when the compound was tested against cell lines engineered to be dependent on either BCL-2 or BCL-X_L_, with substantially lower activity against the BCL-X_L_ dependent cells.

Evaluation in mouse xenograft models of ALL and B cell lymphoma led to significant tumour growth inhibition following oral dosing at 100 mg kg^−1^ over 21 days, with no weight loss observed.^334^ It was also platelet sparing, with a far lower EC_50_ against human platelets than **navitoclax** (5.5 μM compared to 83 nM), and with minimal effects at high doses (100 mg kg^−1^) in dogs. Whilst **venetoclax** shows a drop in drug-likeness, this improved selectivity was the key to its clinical progression.

**Venetoclax** was enrolled in a phase I trial for patients with refractory CLL.^334–336^ The first patients receiving doses exhibited such a rapid reduction in circulating tumour levels that they developed laboratory tumour lysis syndrome (TLS), indicating such extensive tumour cell death that the cellular debris could not be cleared effectively. This was a significant safety concern, and so the dosing regimen was designed to slowly increase over several weeks to the desired dose. At the end of the phase I trial, **venetoclax** had an acceptable safety profile and 79% of patients had displayed objective responses, with 20% achieving complete remission. A phase II study in a larger cohort reported 79.4% of patients achieving objective responses, with 8% in complete remission.^337^ As a result, it was granted breakthrough designation by the FDA and subsequently approved for the treatment of CLL patients with the 17p deletion in September 2016,^338^ which was extended to all patients with CLL or small lymphocytic lymphoma in May 2019. It was also approved in November 2018 as part of a combination treatment for AML.

## Conclusion

There is a rich history of chemical probes informing the development of clinical candidates.^339^ Selective probes for the two cannabinoid receptors, CB1 and CB2, enabled precise investigation of the contribution of each to analgesic and psychotropic effects, eventually informing a decision on targeting CB2 for the treatment of chronic pain.^340^ Probes for members of the dipeptidyl-peptidase (DPP) family of proteins identified toxicity associated with inhibition of some members, but showed that selective inhibition of DPP4 was an effective therapy for the treatment of type-2 diabetes.^341^ This eventually led to the discovery of sitagliptin. A probe for the dual orexin receptor helped guide understanding of the relationship between pharmacokinetics and target-engagement, informing relevant clinical exposure targets.^342^ These studies directly led to the discovery of suvorexant as a treatment for insomnia.

Investigations of target viability, safety, and translation have all been enabled by chemical probes. In addition, obvious structural inheritance highlights the importance of chemical probes in producing drug leads. The probes described (summarised, Table 1) were often first-in-class compounds to interrogate the biology of disease-relevant targets. In the case of **(+)-JQ1** (BET bromodomain probe, Fig. 1), it went on to directly inspire several structurally near-identical clinical candidates, with some showing incredible promise. Both **PFI-1** (BET bromodomain probe, Fig. 7) and **CBP-30** (CBP/p300 probe, Fig. 8) identified pharmacophores that robustly mimicked endogenous substrates and were recapitulated in potential drugs, with the structure of **CBP-30** also being imitated to great effect. **EPZ00477** and **SGC0946** (DOT1L probes, Fig. 11) identified a novel binding mode to impart exquisite selectivity for a target, which was duly employed for clinical benefit. **UNC0638** (G9a/GLP probe, Fig. 14), **EPZ005687** (EZH2 probe, Fig. 16), **EPZ020411** and **MS-023** (type I PRMT probes, Fig. 21) identified key SAR around diverse scaffolds that informed further development, with **EPZ005687** in particular inspiring a molecule that went on to be approved. **EPZ015666** and **LLY-283** (PRMT5 probes, Fig. 24 and 25, respectively) probed the same protein with different binding modes, providing multiple approaches to inhibiting disease-relevant targets. **LLY-283** is also an example of how academic probe development can lead to the discovery of structures remarkably similar to clinical candidates, independent of any collaboration. **GSK′481** (Fig. 27) opened the door to translating pharmacological investigation of necroptosis *in vivo*, whilst **GW4064** (Fig. 30) was a pioneering molecule that identified a key pharmacophore for targeting the FXR. Finally, the development of **ABT-737** (BCL-2 probe, Fig. 34) was a medicinal chemistry tour de force that resulted in a devastatingly effective drug, an example of the benefit of initial research into notionally “undruggable” targets with chemical probes. In general, probes have good drug-likeness scores^14^ but these improve following drug development, with most showing a modest increase in score compared to the probes. Overall, the drugs have much better pharmacokinetics compared to the probes, as we have shown for the examples.

**Table 1:** A summary of the chemical probes described in this review, along with their targets, and the drugs derived from their structures

Probe	Target	Drugs	Indication
**JQ1**	BET bromodomains	I-BET762 (phase I/II)	AML/breast cancer
		OTX015 (phase I)	Glioblastoma
		CPI-0610 (phase III)	Myelofibrosis
**PFI-1**	BET bromodomains	ABBV-075/mivebresib (phase I)	Solid tumours
**CBP-30**	CBP/p300	CCS1477 (phase I)	Prostate cancer
**EPZ004777/SGC0946**	DOT1L	Pinometostat (phase I/II)	MLL-rearranged leukaemias
**UNC0638**	G9a/GLP	EPZ035544 (preclinical)	Sickle cell anaemia
**EPZ005687**	EZH2	GSK126 (phase I)	Haematologic/solid tumours
		Tazemetostat (EPZ-6438) – approved	Advanced epithelioid sarcoma
		CPI-1205 (phase Ib/II	Lymphoma/prostate cancer
**EPZ020411/MS-023**	Type I PRMTs	GSK3367815 – phase I	Solid tumours/DLBCL
**EPZ015666/EPZ015866**	PRMT5	GSK3326595 – phase I/II	Solid tumours/lymphoma
**LLY-283**	PRMT5	JNJ64619178 – phase I	Solid tumours/lymphoma
**GSK′481**	RIPK1	GSK2982772 – phase I	Psoriasis
**GW4064**	FXR	CIlofexor (GS-9674) – phase II	Primary sclerosing cholangitis
		LY25621755 (TERN-101) – phase II	NASH
		Tropifexor (LJN452) – phase II	PBC/NASH
**ABT-737**	BCL2	Navitoclax – phase II	Solid tumours/CLL/SCLC
		Venetoclax – approved	CLL

Beyond their structures, these compounds also represent a potentially transformative approach to drug discovery. The open access model that led to **(+)-JQ1** being widely shared across diverse sections of the research community drove innovation,^343^ leading to a large number of patents being filed in relation to the scaffold. This is a model the SGC has consistently adopted with its probe collection,^7^ which will hopefully continue to inspire clinical candidates and go some way to attenuating the high attrition rate of new drugs.^344,345^ Target 2035^12^ is thus not only a means of probing the entire proteome, but also open access to thousands of molecules that may provide the blueprints for a raft of life-changing therapeutics.

## Conflicts of interest

There are no conflicts to declare.

## Supplementary Material
